# Artificial intelligence and thermodynamics help solving arson cases

**DOI:** 10.1038/s41598-020-77516-x

**Published:** 2020-11-25

**Authors:** Sander Korver, Eva Schouten, Othonas A. Moultos, Peter Vergeer, Michiel M. P. Grutters, Leo J. C. Peschier, Thijs J. H. Vlugt, Mahinder Ramdin

**Affiliations:** 1 Engineering Thermodynamics, Process and Energy Department, Faculty of Mechanical, Maritime and Materials Engineering, Delft University of Technology, Leeghwaterstraat 39, 2628CB Delft, The Netherlands; 2 Netherlands Forensic Institute, P.O. Box 24044, 2490AA The Hague, The Netherlands

**Keywords:** Chemical engineering, Physical chemistry, Computational science

## Abstract

In arson cases, evidence such as DNA or fingerprints is often destroyed. One of the most important evidence modalities left is relating fire accelerants to a suspect. When gasoline is used as accelerant, the aim is to find a strong indication that a gasoline sample from a fire scene is related to a sample of a suspect. Gasoline samples from a fire scene are weathered, which prohibits a straightforward comparison. We combine machine learning, thermodynamic modeling, and quantum mechanics to predict the composition of unweathered gasoline samples starting from weathered ones. Our approach predicts the initial (unweathered) composition of the sixty main components in a weathered gasoline sample, with error bars of ca. 4% when weathered up to 80% w/w. This shows that machine learning is a valuable tool for predicting the initial composition of a weathered gasoline, and thereby relating samples to suspects.

## Introduction

Understanding the weathering of gasoline due to fire is crucial in forensic investigation of arson^1^. In such cases, DNA-comparison is often not possible and forensic researchers are deemed to look for other ways to collect evidence against arson suspects. A comparison of the composition of a weathered gasoline sample taken at the crime scene with original (unweathered) gasoline samples found in the possession of a suspect can provide evidence which links the suspect to the fire scene. Due to the refining process, neat gasoline has a very characteristic composition fingerprint, which can be used by forensic scientists to discriminate between different gasoline samples. For weathered samples this is far from trivial, because: (1) the composition of gasoline changes significantly upon weathering, (2) gasoline is a complex mixture of hundreds of components (all with different weathering behavior), and (3) the weathering process may be affected by many factors such as the evaporation temperature, fire extinguishing water, preferential adsorption on the substrate (matrix effects), and microbial degradation^1,2^. Despite these possibly interfering effects, gasoline in fire debris samples is often sufficiently preserved to make a forensic comparison possible. Unfortunately, this composition cannot be directly linked to the composition of the original (unweathered) sample, because of weathering effects. For this reason, a strategy needs to be developed to successfully compare weathered and unweathered samples. Different approaches have been proposed to overcome this complication^3^. Researchers have tried to tackle the problem without using an explicit evaporation model, for instance by using statistical methods such as principal component analysis (PCA)^4–6^, linear discriminant analysis (LDA)^4,6^, canonical variate analysis (CVA)^6^, hierarchical cluster analysis (HCA)^5^ or by covariance mapping^7^. One proposed method is a likelihood ratio approach^8^, implicitly using a simple evaporation model. Other studies have used explicit evaporation models based on Raoult’s law^9,10^ or by using gas chromatographic retention data^11–14^, and other experimental methods^15,16^. All these methods suffer from major drawbacks, e.g., only a limited number of components can be dealt with, the methods cannot be applied to highly weathered samples, large experimental data sets are required, and most importantly, the methods cannot predict the initial (unweathered) composition of a weathered sample. This is highly important as a sample found at a suspect is nearly always unweathered. To make a quantitative comparison between weathered and unweathered samples, we propose a method which combines machine learning, advanced thermodynamic modeling, and quantum chemical calculations. For the first time, this allows accurate predictions of the initial (unweathered) composition of a weathered sample. Our method can be generalized to any number of components and is able to discriminate between samples of different degree of weathering. Additional complicating effects such as preferential adsorption and fire extinguishing media are not considered here, as these are highly case specific. The artificial neural networks (ANNs), which are machine learning algorithms, were trained on field data from the Netherlands Forensic Institute (NFI). The proposed method is applied to model composition changes of gasoline samples assuming that the effects of preferential adsorption, pyrolysis, microbial degradation, and eventual fire extinguishing media are negligible. We will investigate this ideal case in which composition changes are solely due to evaporation. These simplified conditions are important for model development and testing, because models that fail under these conditions will also fail when all interfering effects are included. The simplified conditions represent a limiting case in practice, for example, when gasoline is ignited on a non-adsorbing ground.

**Figure 1:**
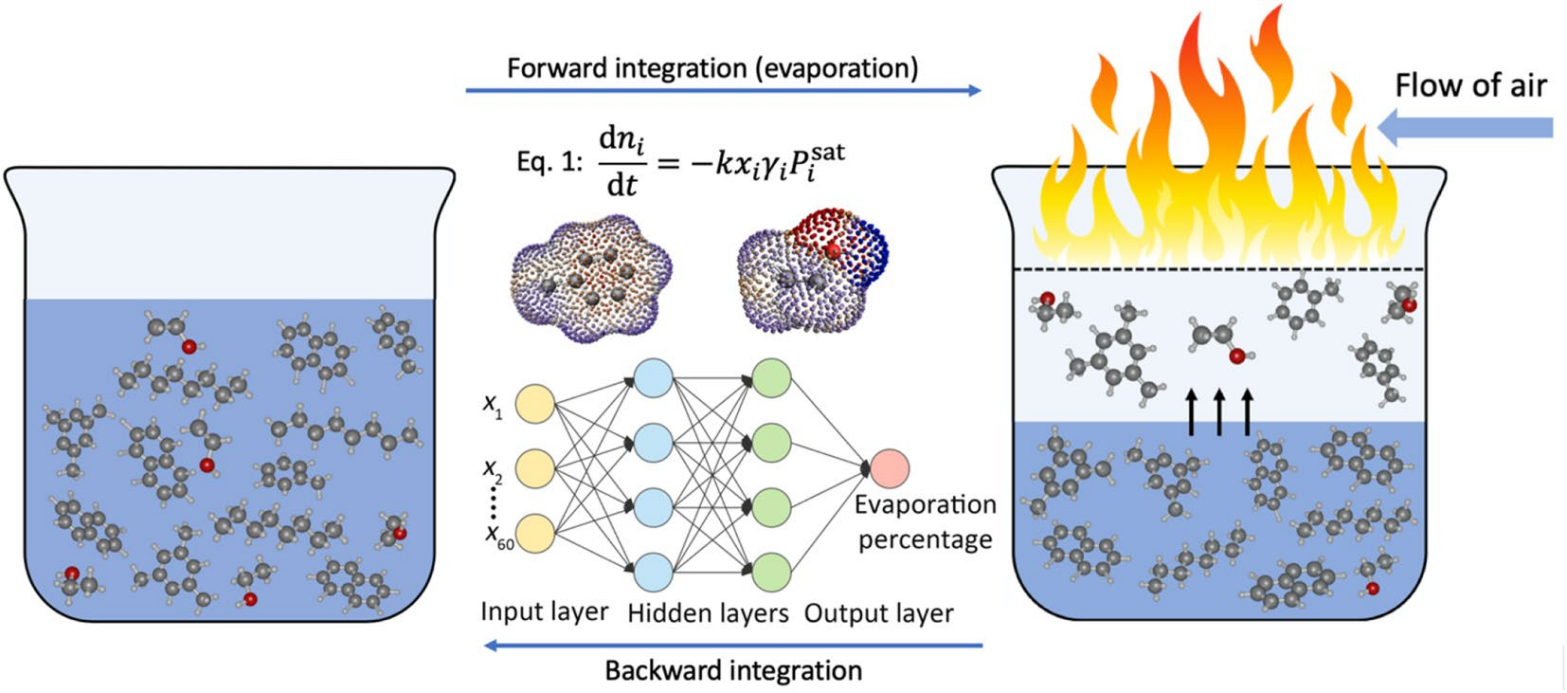
Schematic representation of the weathering process and the used modeling approach. Left: an unweathered (original) gasoline sample, which is found in possession of a suspect. Right: the composition changes or weathering of a gasoline mixture due to fire. A combination of thermodynamic modeling, quantum chemical calculations, and machine learning is used to link the composition of the weathered and unweathered samples. The weathering process is described by Eq. (1), which is based on the gamma-phi approach of vapor–liquid equilibrium calculations. Forward integration of Eq. (1) is used to predict the composition of a weathered sample starting from the original sample. Backward integration of Eq. (1) is used to obtain the composition of the original gasoline starting from a weathered sample. In Eq. (1), n_i_, x_i_, γ_i_, and P_i_^sat^ are the number of moles, the mole fraction, the activity coefficient, and the saturated vapor pressure of component i in the liquid phase, respectively. Activity coefficients of the multicomponent mixture were obtained from the COSMO-RS model^17^. Artificial neural networks (ANNs) were trained on field data and used to predict the evaporation percentage or the degree of weathering for a given composition.

In Fig. 1, a schematic overview of the problem and the proposed method is illustrated. Neglecting the interfering effects described above, the problem can be reduced to the calculation of the evaporation of gasoline, the determination of the degree of weathering, and the backward or forward tracking of the composition change of weathered and unweathered samples. In backward tracking, the challenge is to recover the initial composition of a sample starting from a weathered sample. In forward tracking, one starts with the original sample and records the composition change as the degree of weathering is increased. The aim is to devise a method that can model the evaporation of gasoline considering the nonideality of the multicomponent mixture arising from the presence of polar compounds. We propose a thermodynamic model supplemented with quantum chemical calculations and machine learning to obtain a quantitative understanding of the weathering process of gasoline.

The thermodynamic model is based on the gamma-phi approach for vapor–liquid equilibrium (VLE) calculations and requires a numerical solution of the differential equation given by Eq. (1) in Fig. 1, which is essentially a modified version of Raoult’s law^18^. In Eq. (1), the following assumptions have been made; (1) the liquid phase is well-mixed and the evaporation process is not limited by mass transfer in the liquid, (2) the gas phase is assumed to be ideal, which is a reasonable and common assumption at standard conditions, (3) the mass transfer coefficient in the gas phase, *k*, is equal for all components, and (4) the temperature of the liquid is constant during the evaporation process. As the evaporation time is irrelevant, *k* and *t* can be combined to yield a dimensionless variable *ϕ*. In principle, the mass transfer coefficient is component dependent, but it is extremely challenging (almost impossible) to measure it for such a complex mixture, and it is not necessary for the present analysis. It is important to note that the assumptions above are crucial to keep the model tractable in absence of any other experimental data. The temperature dependent vapor pressure of gasoline components can be found in databases such as NIST, DIPPR, and DDBSP^19–21^. The activity coefficient is a measure for the nonideality of a system, where γ = 1 means an ideal solution, and depends on the composition and temperature of the mixture. The activity coefficients required to describe the nonideality of gasoline were obtained from the COSMO-RS model, which relies on quantum chemical computations of the individual components in the system^17,22^. We will show that activity coefficients have a strong influence on the weathering of gasoline in the presence of polar components such as ethanol and ethers (e.g., methyl-*tert*-butylether). By integrating Eq. (1) either forward or backward in time, we can model the evaporation as well as back trace the original (unweathered) composition of a weathered sample. In the latter case, there is a serious practical complication: for a given weathered sample one usually does not know the degree of evaporation, so one does not know when to stop the backward integration of Eq. (1). We overcome this complication by using artificial neural networks (ANNs), which can be trained to recognize patterns in data and solve highly non-linear problems. The composition change of a multicomponent mixture is correlated with the degree of weathering in a highly non-linear way. For example, the most volatile components are completely evaporated at relatively low degree of weathering, while the least volatile components remain in the mixture up to high degree of evaporation. This correlation is related to the properties of the molecules that comprise the mixture, which depend on composition and temperature, making it a highly non-linear process. Due to the complexity of gasoline mixtures, such a correlation is difficult to observe with bare eyes, but machine learning is perfectly suited for this task^23^. Like the human brain, ANNs can be trained to recognize patterns in data, e.g., evaporation data of gasoline mixtures^24^. Here, we have trained the ANN on a large dataset of evaporation curves, starting from 459 gasoline samples collected in 2011 at petrol stations in the Netherlands and analyzed by the NFI. In our model, we have used a mixture of 60 main components of gasoline, which comprises the most abundant components observed in arson samples. This ANN model enables us to predict the degree of evaporation in weathered gasoline samples with differences of ca. 3%, for gasolines that are evaporated up to 80 w%. The composition of individual components in the predicted initial (unweathered) samples is estimated with a maximum deviation of 4% for the most volatile components. The intervariation of components in unweathered gasoline is much larger than 4%. The main conclusion from these results is that all components, including the volatile ones, can now be included for an effective forensic gasoline comparison.

## Results

**Figure 2:**
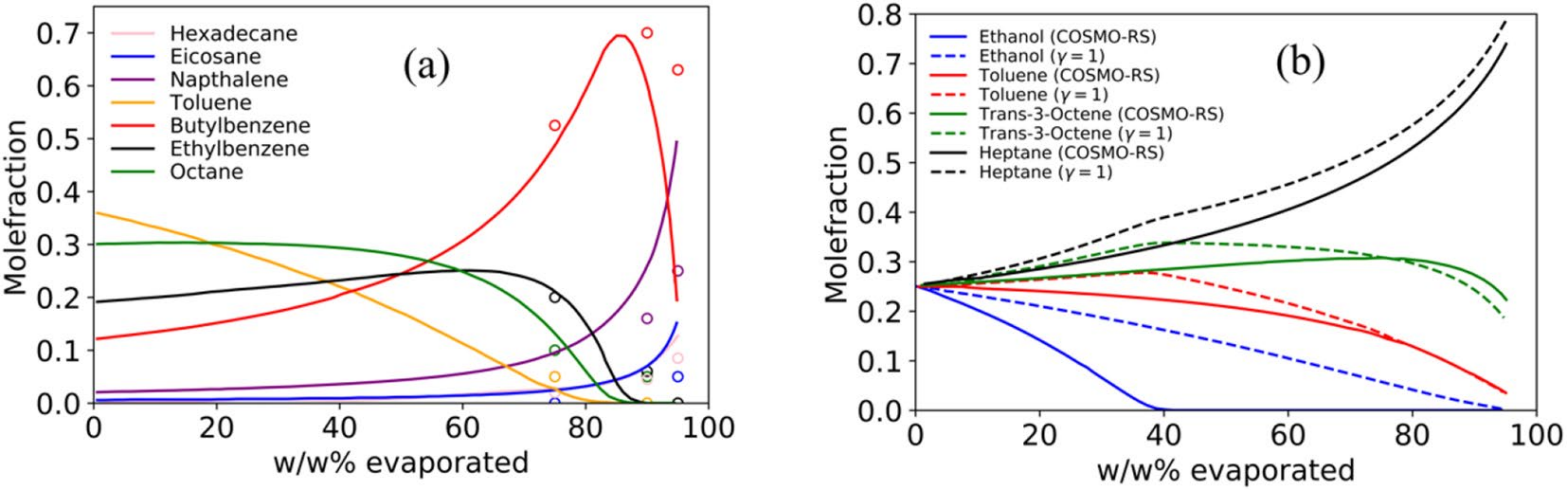
Effect of weathering degree (i.e., % evaporated) and activity coefficients on the composition change of synthetic gasoline-like samples. (**a**) Composition change of a seven component (hexadecane, eicosane, naphthalene, toluene, butylbenzene, ethylbenzene, and octane) synthetic nonpolar gasoline mixture as a function of the degree of weathering at 298.15 K. Lines are modeling results and symbols are experimental data. Note that the activity coefficients have a small effect on the evaporation behavior of this nonpolar mixture (the lines of γ = 1 coincide with the lines computed from COSMO-RS). (**b**) The effect of activity coefficients on the evaporation behavior of a four component (ethanol, toluene, trans-3-octene, and heptane) synthetic gasoline-like mixture at 298.15 K. Dashed lines are for ideal evaporation (γ = 1) and the solid lines show non-ideal behavior with activity coefficients computed from COSMO-RS.

The proposed thermodynamic modeling approach is first tested for the evaporation of a seven-component nonpolar mixture representing an artificial gasoline. Figure 2a shows the compositional change of the mixture as a function of the weathering degree. The model correctly describes the evaporation behavior of this mixture compared with experimental data from literature. The following observations are made: (1) the composition change of the sample strongly depends on the weathering degree; (2) the low volatile components accumulate in the residue as more gasoline is evaporated; (3) the activity coefficients seem to have a small effect, which is not surprising for such a nonpolar mixture; and (4) as shown in the Supplementary Information, temperature effects tested up to 330 K are very small. However, real gasoline contains several polar components like ethanol and/or ethers, which drastically change the evaporation process. To illustrate the effect of polar compounds on the weathering of gasoline, we have used a synthetic four component gasoline-like mixture containing ethanol, toluene, trans-3-octene, and heptane. In Fig. 2b, a clear effect of ethanol on the evaporation behavior is observed. The evaporation behavior of ethanol and the other components strongly depends on the activity coefficients. For example, ethanol is completely evaporated at around 90 wt% when the mixture is considered ideal, and at 40 wt% when the more realistic activity coefficients from COSMO-RS are used. Therefore, it is crucial to consider the nonideality of the mixture caused by polar-nonpolar interactions to correctly describe the evaporation process. This requires the computation of activity coefficients, which are functions of composition and temperature. Activity coefficients higher than one (positive deviation from Raoult’s law) or lower than one (negative deviation from Raoult’s law) will increase or decrease the vapor pressure of the components in the mixture.

The large number of components in gasoline prohibits the use of classical activity coefficient models such as NRTL and Wilson, which require a huge amount of experimental data for fitting binary parameters^25^. Furthermore, these binary parameters cannot account for higher order interactions that are present in multicomponent mixtures. We have used the COSMO-RS model^17^, which is based on quantum chemistry, to predict the activity coefficients of the mixtures. COSMO-RS uses a quantum chemical calculation to obtain the screening charge density (i.e., sigma-profiles) of molecules from which thermodynamic properties such as the activity coefficient are derived^17,22^. We note that in absence of any experimental data, the COSMO-RS method is currently the state-of-the-art approach to compute activity coefficients of multicomponent mixtures^26^. Typical errors in the activity coefficients computed from COSMO-RS compared to experiments are < 10% and < 30% for nonpolar and polar compounds, respectively.

**Figure 3:**
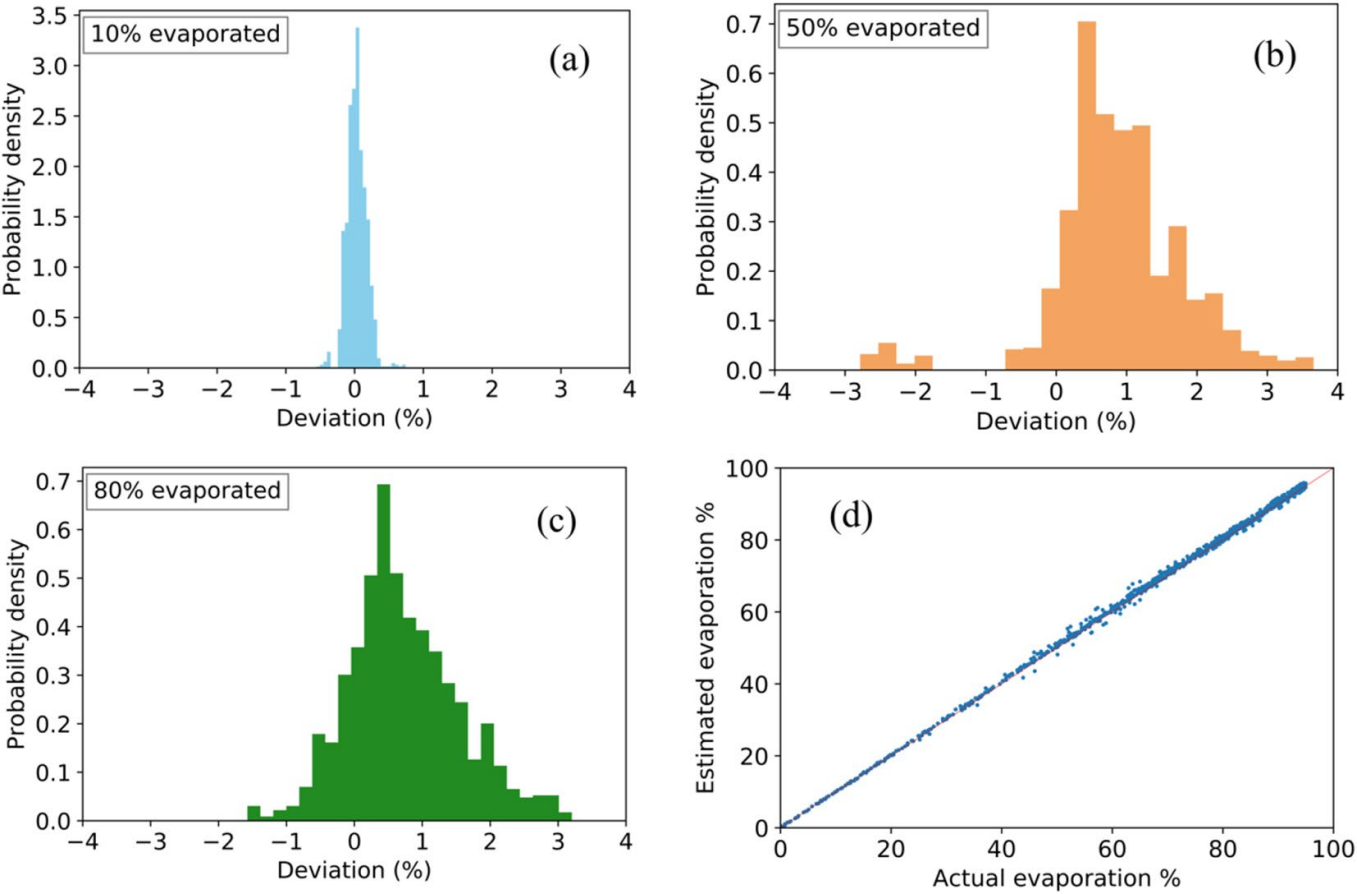
Performance of ANNs for predicting the degree of evaporation for samples evaporated up to (**a**) 10%, (**b**) 50%, and (**c**) 80% at 298.15 K. The deviation percentage is defined as the difference between the actual degree of evaporation and the estimated degree of evaporation by the ANN. (**d**) Scatterplot of the actual evaporation percentage and the evaporation percentage estimated by the ANN model. The data points correspond to degrees of evaporation between 0 w% and 95 w%.

We now have all the required tools to model the evaporation of real gasoline samples. The direct use of the thermodynamic model requires the composition of the weathered or unweathered sample and a stopping criterion for the forward or backward integration. Starting from a weathered sample requires backward integration to successfully compare the unweathered sample. Similarly, starting from an unweathered sample requires forward integration (i.e., evaporation) to compare the composition of the weathered sample. This requires knowledge on the degree of weathering, which is unknown for samples collected at a crime scene. To overcome this complication, we use artificial neural networks to estimate the degree of weathering from a given input composition, which were obtained from field data collected and analyzed by the NFI. In total 459 gasoline samples were collected at different petrol stations throughout the Netherlands and analyzed for 60 main components, see the Supplementary Information for details. The thermodynamic model was then used to perform the evaporation experiments of these sixty-component gasoline samples. The full evaporation curves of 400 samples (each containing 6000 data points) were used to train the ANNs. The remaining 60 gasoline samples were used to validate the trained model. The input to the ANNs are the mole fractions of all 60 components and the predicted variable is the degree of weathering (i.e., evaporation percentage). After training, 360,000 data points from the 60 remaining gasoline samples were used to evaluate the accuracy of the ANN model. In Fig. 3a–c, the performance of the ANN for estimating the degree of weathering for 10%, 50%, and 80% evaporated samples is shown. In Fig. 3d, a scatterplot of the actual evaporation percentage and the estimated evaporation percentage is shown. The histograms show that the ANN is very accurate in predicting the degree of weathering for samples evaporated up to 10%, showing deviations up to 0.5%. The accuracy of the ANN model drops slightly as the degree of weathering is increased from 10 to 50% and 80%, which results in a deviation of around 3% for the highest degree of evaporation. This is expected as experiments indicate that it is more challenging to track the composition of heavily weathered samples. The reason for this is that an increasing number of components are almost completely evaporated as the degree of weathering is increased, leading to larger errors when predicting the original composition. Similarly, the ANN model requires many more training data for heavily weathered samples than for slightly weathered samples. Overall, the accuracy of the ANN model in predicting the evaporation percentage of a randomly selected sample not part of the training set is within 3%.

## Backward tracking of evaporation

**Figure 4:**
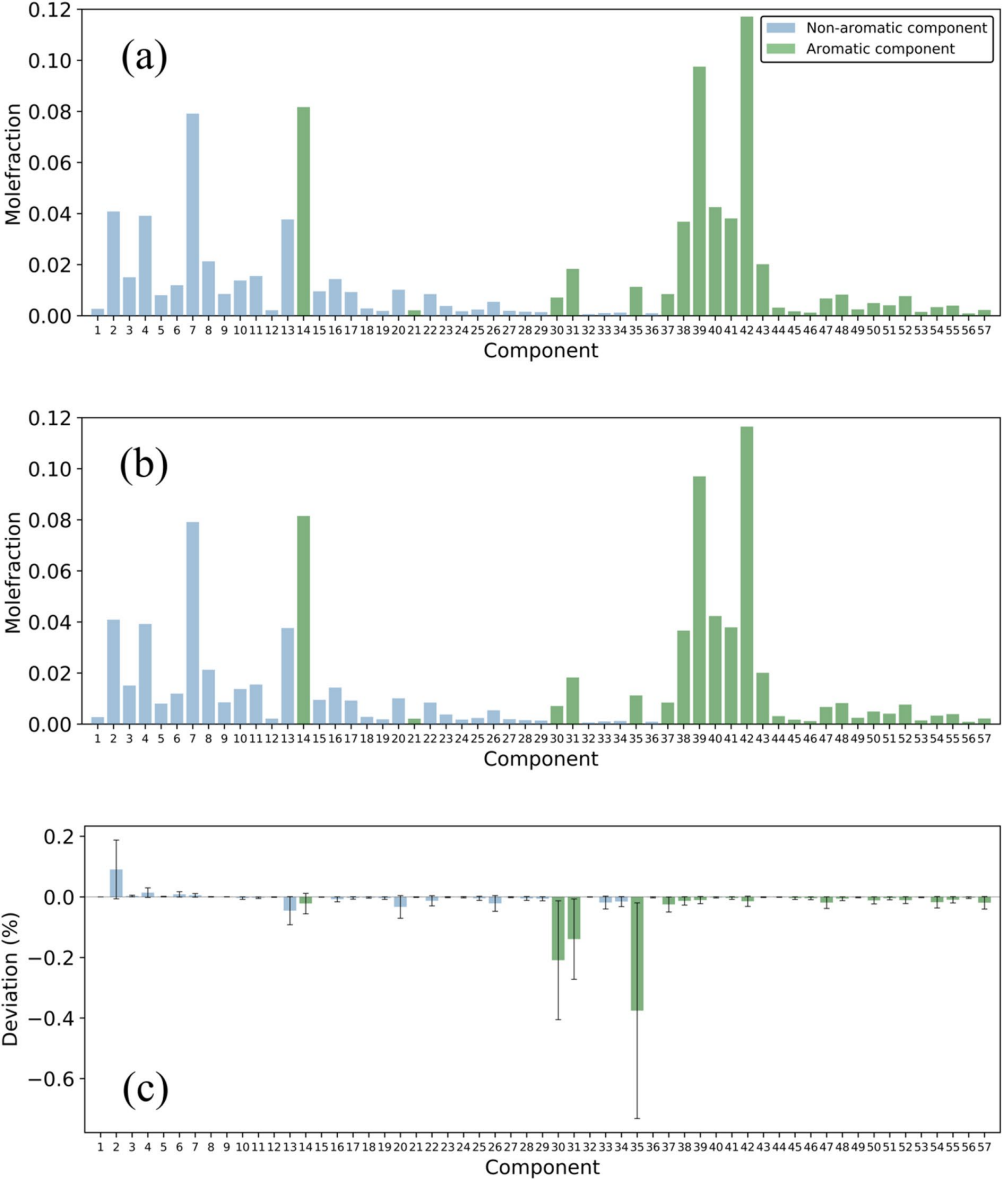
Back tracing of the initial composition of a 50% weathered sample using the ANN model. (**a**) the composition of an unweathered sample, (**b**) the initial composition of a 50% weathered sample estimated by the ANN, and (**c**) the deviation between the estimated composition and the actual composition. The names of the components can be found in the Supplementary Information. The error bars show the 95% confidence interval. The deviations for the oxygenated components (58, 59, 60) are less than 4% (not shown here).

In the previous section, we have used an ANN model to predict the degree of weathering from a given composition. The goal is to predict the original composition of a sample with an unknown degree of weathering. By first estimating the degree of weathering using the ANN model and then performing the backward integration of Eq. (1), we are able to track the initial composition of the sample. Figure 4a,b show a comparison of the composition of an unweathered sample and the predicted composition of a 50% weathered sample using the ANN model. In Fig. 4c, one can see the corresponding deviation in the predicted composition and the actual composition. The maximum deviation is observed around 4% for the oxygenated components. For the other, aromatic and non-aromatic, compounds the deviation is less than 1%. This means that it is more challenging to accurately predict the composition change of volatile compounds. Since the intervariation between compositions of gasoline is much larger than 4%, a forensic comparison including volatile compounds now becomes possible. This makes such a comparison much more effective.

## Discussion

Tracking the composition change of a weathered gasoline sample is a challenging problem, which is of utmost importance for forensic investigators dealing with arson crimes. The comparison of the composition of a (weathered) sample from a crime scene and an unweathered sample found in possession of a suspect is complicated by: (1) the complexity of gasoline mixtures, and (2) the interfering effects of evaporation, preferential adsorption, combustion, pyrolysis, microbial degradation, and fire extinguishing water. By assuming that the composition change of a gasoline sample is solely due to evaporation, and thus neglecting the other interfering effects, allowed us to predict the weathering process with a thermodynamic model supplemented with quantum chemical calculations and machine learning tools. To describe the evaporation process, we demonstrated that it is crucial to consider the nonideality of gasoline mixtures. The activity coefficients were computed from COSMO-RS, which has a typical error of < 10% and 30% for nonpolar and polar compounds, respectively. This accuracy is acceptable for the current application, because the results show that errors in predicted compositions for volatile compounds are less than 4%, which is much lower than the intervariation between gasolines. By training ANNs with field data from the NFI, we were able to estimate the degree of weathering and back trace the composition of the weathered sample. The accuracy of the predictions from the ANN model depends on the degree of weathering, e.g., the deviation in the estimated composition increased from 0.5% to 4% for 10% and 80% weathered samples, respectively. The accuracy of the ANN model can be increased by using more data for the training, especially for highly evaporated samples. Furthermore, the accuracy of thermodynamic model can be improved by using a more accurate activity coefficient model for the highly weathered solutions. The COSMO-RS model is relatively accurate for dilute solutions, but may deteriorate at high concentrations (high degree of weathering). The thermodynamic model based on the gamma-phi method is restricted to non-supercritical conditions. For supercritical conditions, the model will require extrapolation of vapor pressure data or the use of the phi-phi approach for VLE calculations. However, for the current problem, the gamma-phi method is straightforward to use and the expectation is that the performance in the supercritical regime will not be affected that much, since the temperature (tested up to 330 K) seems to have a small effect on the weathering process, see the Supplementary Information.

## Methods

### Thermodynamic modeling

The evaporation process is modeled by iteratively solving Eq. (1). The saturation vapor pressures of the components were taken from literature, while the activity coefficients were computed with the COSMO-RS method. A Python code is used to numerically solve the equation using Heun’s method, which is an improved version of Euler’s method. For each calculation step, the code sends the composition of the mixture to the Amsterdam Density Functional (ADF, version 2018.104) software package, which uses the COSMO-RS method to compute the activity coefficients. 41 out of the 60 components could be found in the ADF database. The remaining 19 components were created in the ADF software package and the COSMO-RS sigma profiles were calculated. The computed activity coefficients from COSMO-RS were then used in the next integration step, which was continued until a desired degree of weathering was achieved.

### Generation of gasoline data for the simulations

Details of collecting gasoline reference samples are provided by Peschier et al.^27^ . The gasoline samples were collected in the period of May–August 2011 from 230 different petrol stations in the Netherlands. In total, 28 different brands were collected from international oil companies (Shell, Esso, BP, Q8, Texaco, Gulf, Total, Avia, Tamoil), regional operating companies (Tango, Tinq, Brand Oil, Firezone), and local sellers. At every petrol stations, gasoline from two fuel pumps was collected, one with an Euro 95 grade gasoline and one with high octane (98 RON) or high-performance grade gasoline (such as Shell V-power, Esso Energy Supreme, BP Ultimate, Total Exellium) leading to a total of 459 gasoline samples.

The chemical composition of the gasoline samples was determined by gas chromatography with flame ionization detection (FID) following the procedure by Vergeer et al.^8^. An Agilent 6890 N gas chromatograph was used, equipped with an FID detector and an Agilent 7683 Series autosampler. The column was a 25 m × 0.20 mm × 0.33 μm methyl silicone (Ultra 1) column. The oven temperature was ramped with 2 °C/min from 50 to 160 °C. An automated procedure, using Chromeleon software, was used to integrate a selection of 60 well resolved peaks in the chromatograms. Mole fractions have been calculated by dividing the peak areas of the components by the number of carbon atoms in the molecule. The oxygenated components were not measured, due to coelution with the solvent. However, as these components influence the evaporation process greatly, they have been added manually, using typical concentrations as applied in gasoline in the Netherlands: 3 w% ethanol, 1 w% MTBE and 1 w% ETBE have been added. After that, the data were normalized so the mole fractions add up to 1.

### Machine learning

Here, we have used a dense neural network, which was integrated in the existing system using TensorFlow. The built-in DNNRegressor estimator was used, combined with the ProximalAdagrad optimizer. The constructed deep neural network consists of 5 hidden layers, which contain 1024, 512, 256, 128 and 64 nodes, respectively. The hyperparameters of the neural network were not systematically optimized. We verified that the accuracy of the neural network predictions did not change when slightly changing the number of neurons in the first hidden layer. The dataset used for training consist of the evaporation curves of 400 gasolines. The remaining 60 gasolines were used for the validation of the trained model. This neural net takes the mole fractions of all 60 components as input. The output is an estimation of the evaporation percentage. After training, 360,000 data points from the 60 remaining gasolines were used to evaluate the accuracy of the model.

## Supplementary information

Supplementary information 1.

Supplementary information 2.

## Data Availability

All data used in this work are provided in the Supplementary Information. Vapor pressure data of all components and compositions for the 459 gasoline mixtures used for training the ANN are provided in an Excel file in the Supplementary Information.

## Code availability

The Python code for the thermodynamic modeling and machine learning will be made available upon reasonable request. The COSMO-RS tool can be obtained from the ADF developers at https://www.scm.com.
