# Supplementary material for: Artificial intelligence and thermodynamics help solving arson cases
Source: Sci Rep. 2020 Nov 25;10:20502. doi: 10.1038/s41598-020-77516-x (PMC7689476; doi:10.1038/s41598-020-77516-x)
Supplement: Supplementary file 1 — Supplementary information 1. [file 41598_2020_77516_MOESM1_ESM.pdf]

Supporting Information for: Artificial intelligence and thermodynamics help solving arson cases

Sander Korver[a], Eva Schouten[a], Othonas A. Moultos[a], Peter Vergeer[b], Michiel M.P. Grutters[b], Leo J.C. Peschier[b], Thijs J.H. Vlugt[a], Mahinder Ramdin*[a]

[a] Engineering Thermodynamics, Process and Energy Department, Faculty of Mechanical, Maritime and Materials Engineering, Delft University of Technology, Leeghwaterstraat 39, 2628CB Delft, The Netherlands

[b] Netherlands Forensic Institute, P.O. Box 24044, 2490AA, The Hague, The Netherlands

* Corresponding author: m.ramdin@tudelft.nl

This Supporting Information contains the following:

- Figures showing the effect of temperature on the evaporation behavior of a sixty component gasoline mixture (Figure S1 to S5)
- Figures showing the deviation in the back traced initial composition of a 50% weathered sample and the unweathered sample (Figure S6 to S44)

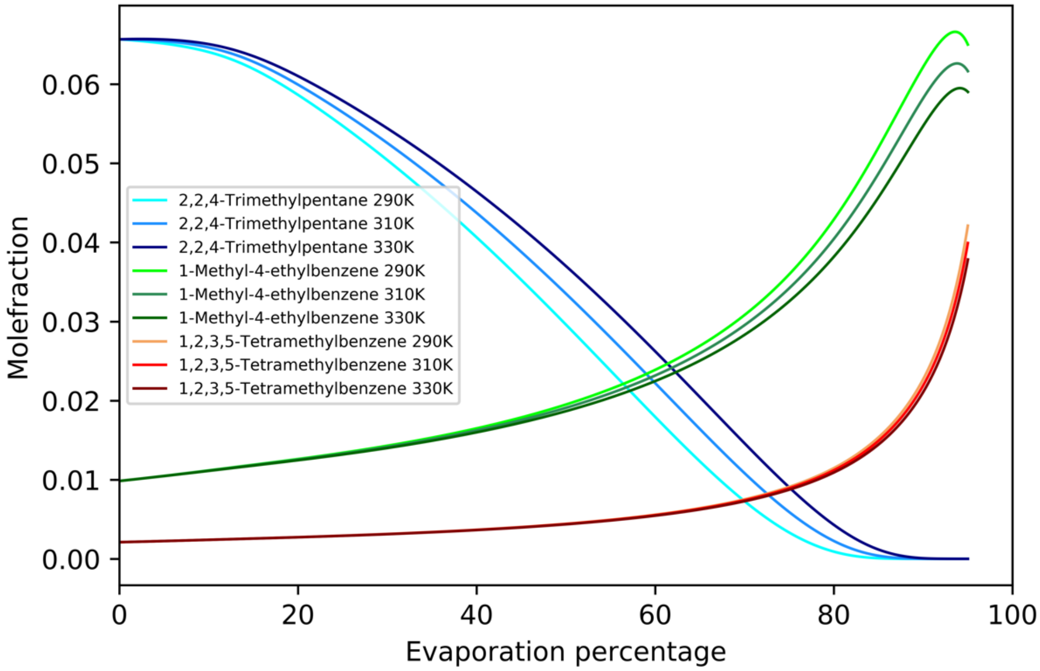

Figure S 1. Effect of temperature on the evaporation of a sixty component gasoline mixture. Evaporation curves of some selected components are shown.

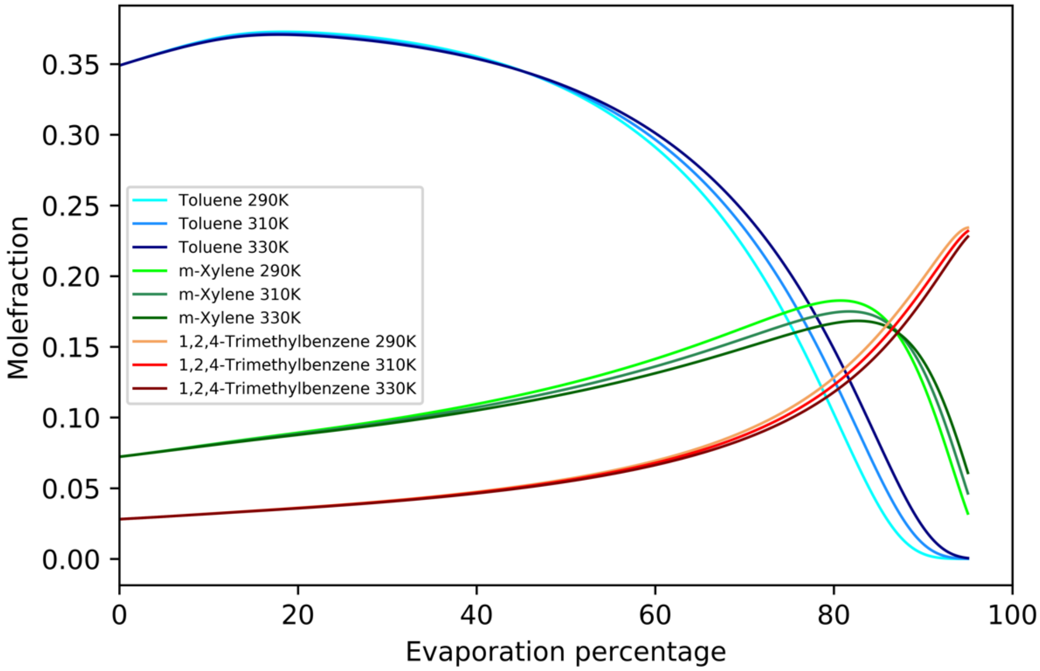

Figure S 2. Effect of temperature on the evaporation of a sixty component gasoline mixture. Evaporation curves of some selected components are shown.

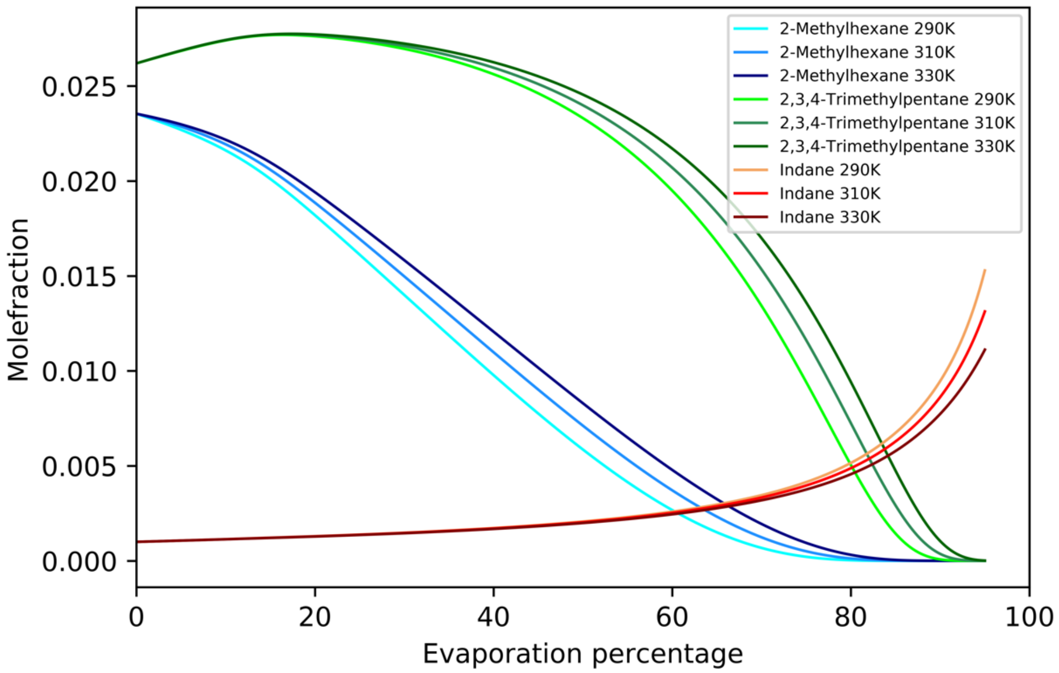

Figure S 3. Effect of temperature on the evaporation of a sixty component gasoline mixture. Evaporation curves of some selected components are shown.

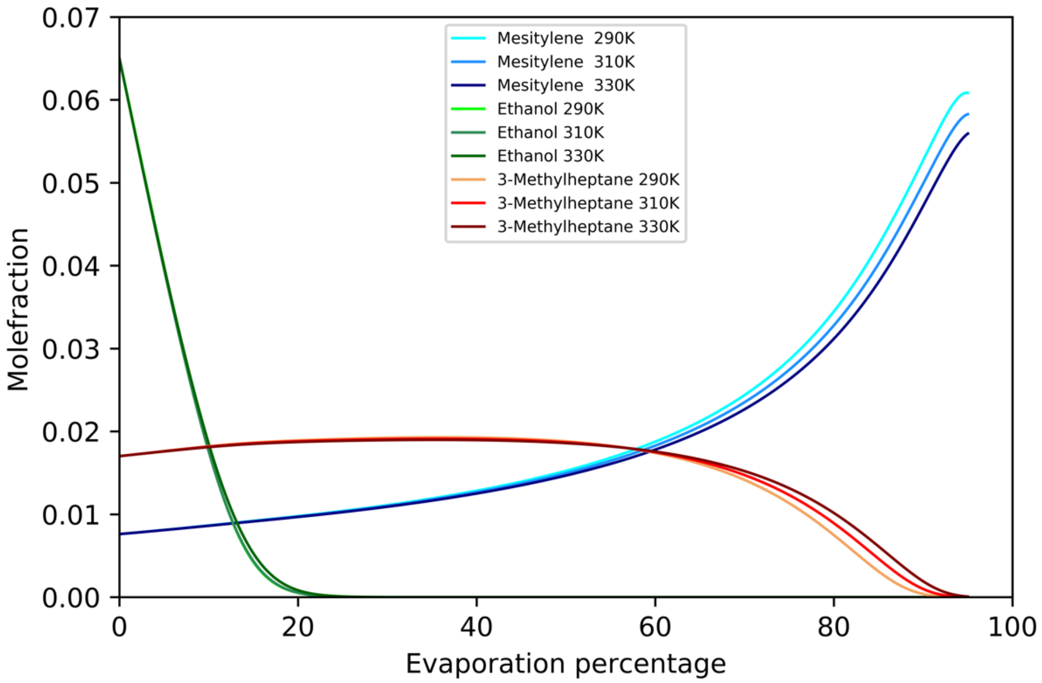

Figure S 4. Effect of temperature on the evaporation of a sixty component gasoline mixture. Evaporation curves of some selected components are shown.

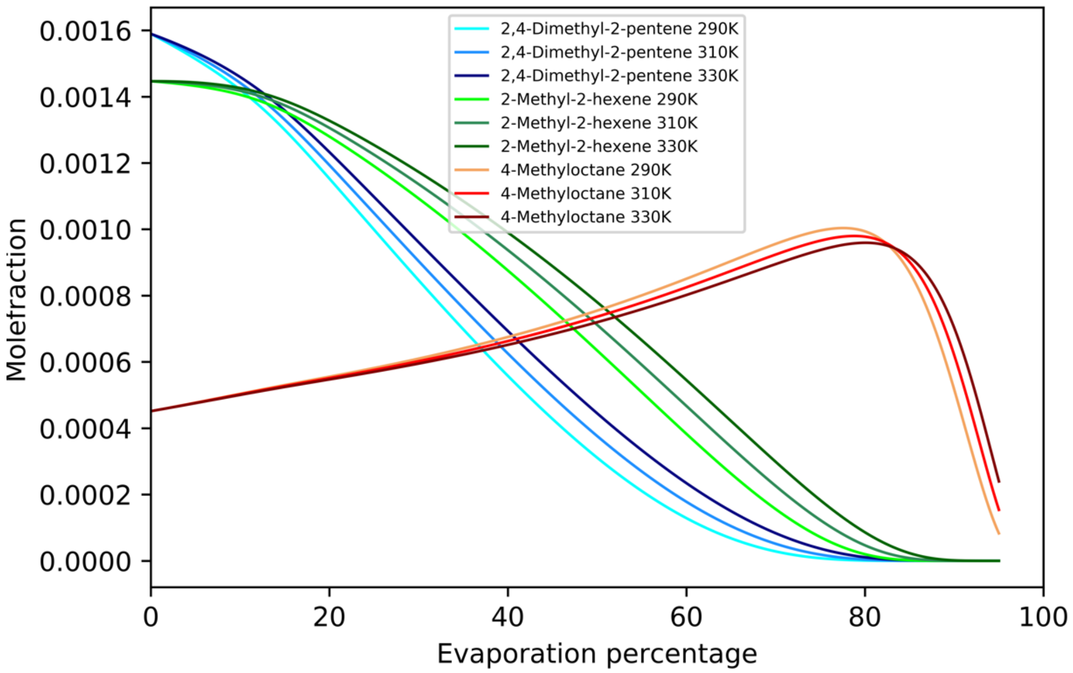

Figure S 5. Effect of temperature on the evaporation of a sixty component gasoline mixture. Evaporation curves of some selected components are shown.

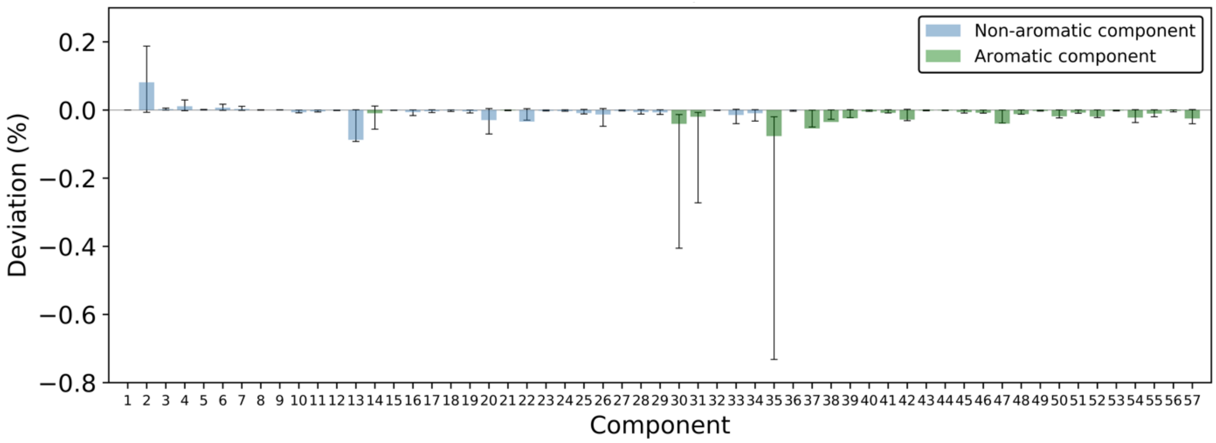

Figure S 6. Prediction of the initial composition of a 50% weathered sample using the ANN and the actual composition. Gasoline sample number: 400.

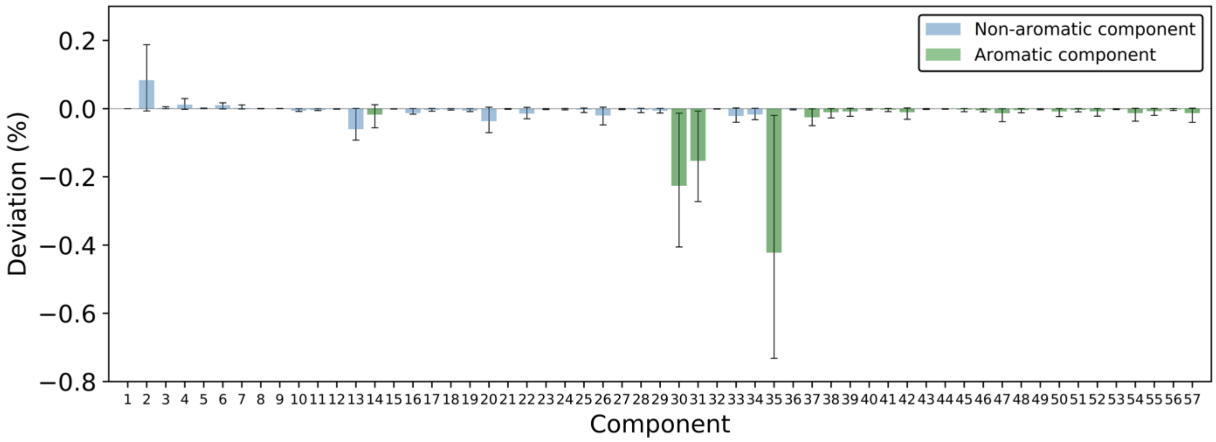

Figure S 7. Prediction of the initial composition of a 50% weathered sample using the ANN and the actual composition. Gasoline sample number: 401.

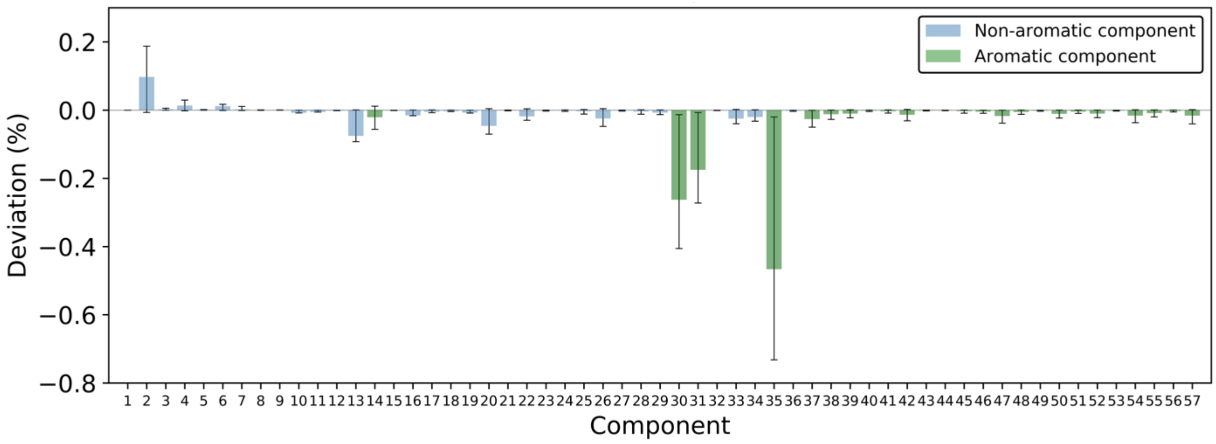

Figure S 8. Prediction of the initial composition of a 50% weathered sample using the ANN and the actual composition. Gasoline sample number: 402.

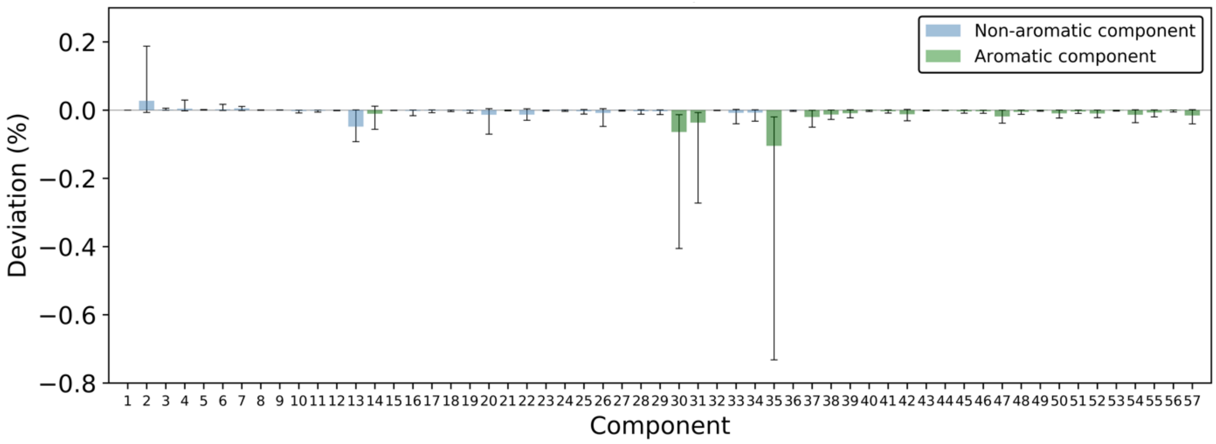

Figure S 9. Prediction of the initial composition of a 50% weathered sample using the ANN and the actual composition. Gasoline sample number: 403.

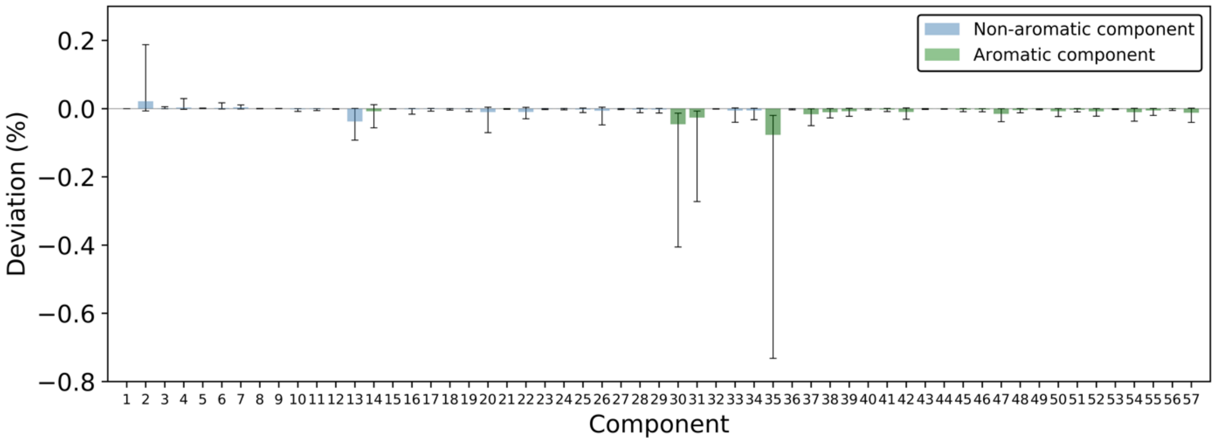

Figure S 10. Prediction of the initial composition of a 50% weathered sample using the ANN and the actual composition. Gasoline sample number: 404.

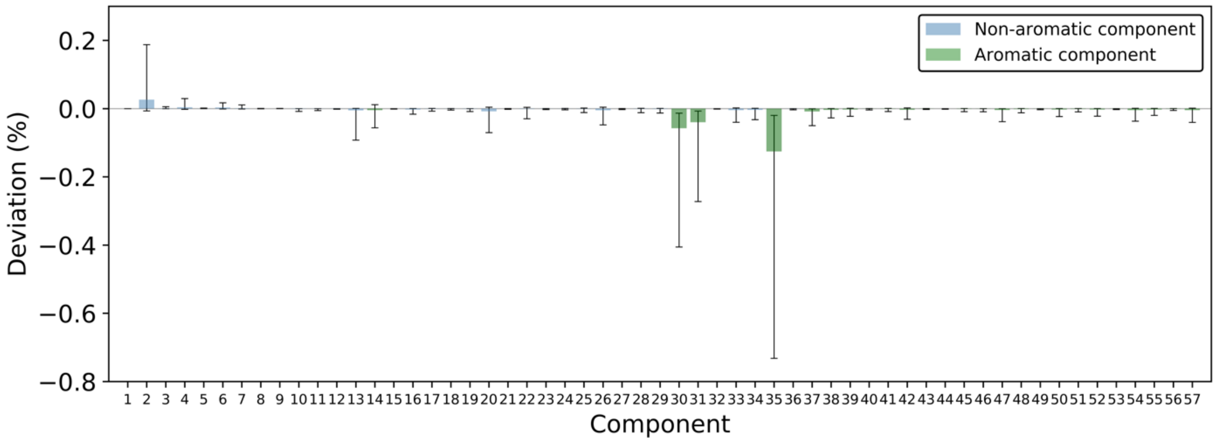

Figure S 11. Prediction of the initial composition of a 50% weathered sample using the ANN and the actual composition. Gasoline sample number: 405.

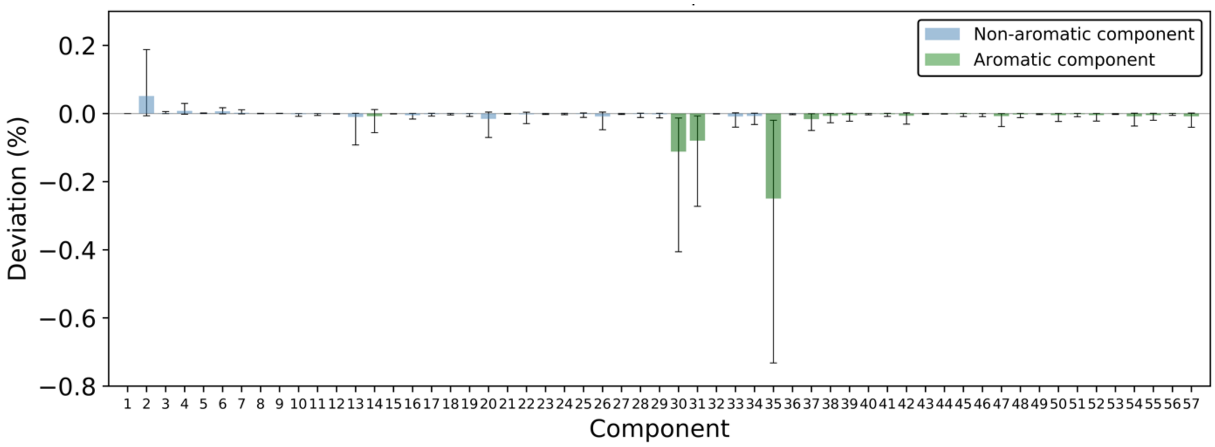

Figure S 12. Prediction of the initial composition of a 50% weathered sample using the ANN and the actual composition. Gasoline sample number: 406.

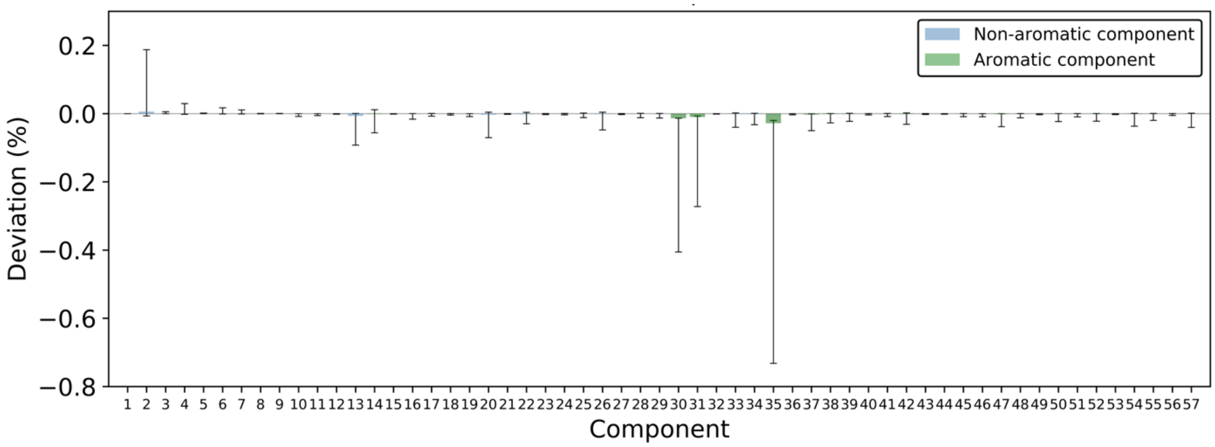

Figure S 13. Prediction of the initial composition of a 50% weathered sample using the ANN and the actual composition. Gasoline sample number: 407.

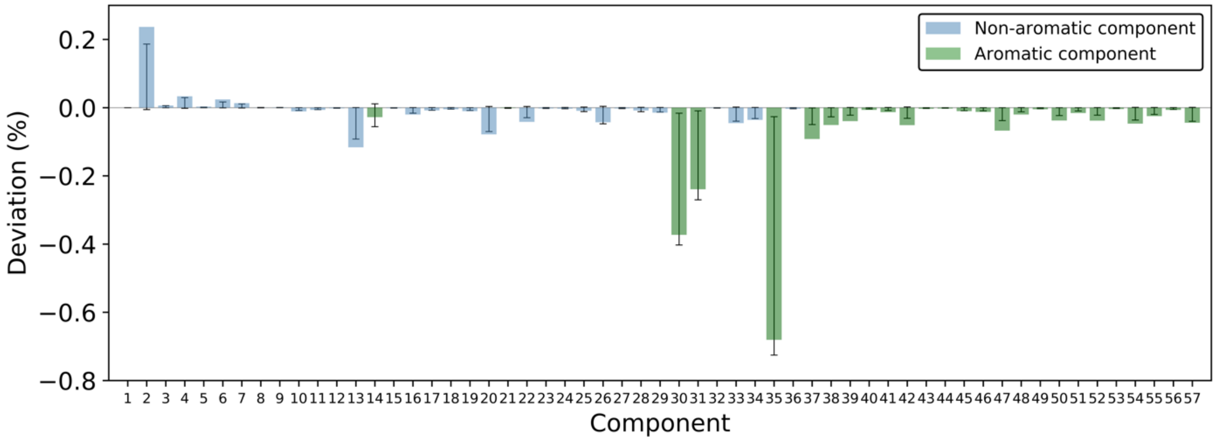

Figure S 14. Prediction of the initial composition of a 50% weathered sample using the ANN and the actual composition. Gasoline sample number: 408.

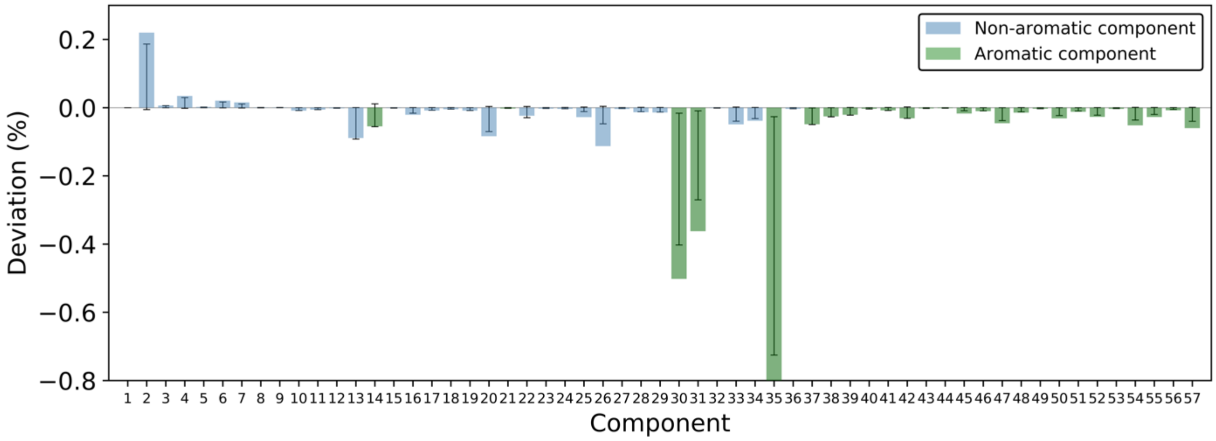

Figure S 15. Prediction of the initial composition of a 50% weathered sample using the ANN and the actual composition. Gasoline sample number: 409.

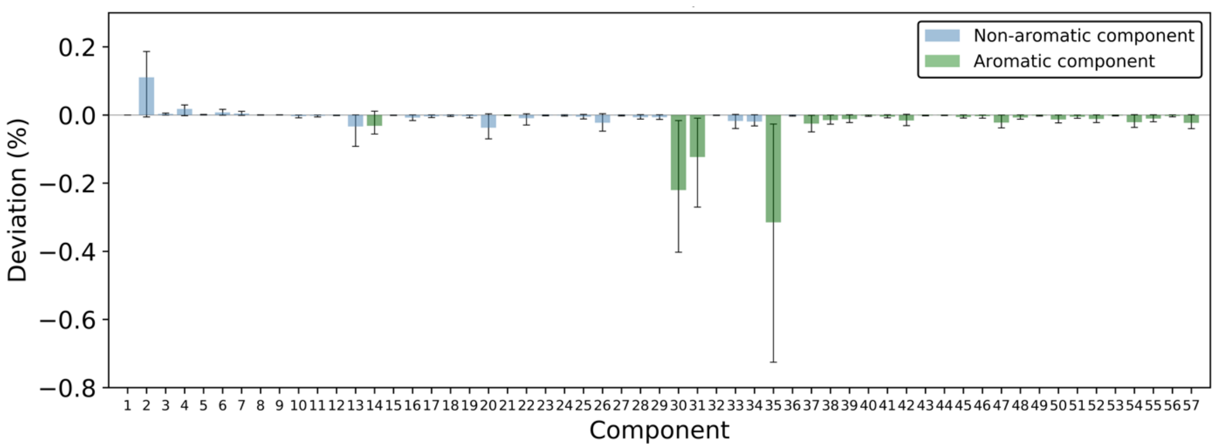

Figure S 16. Prediction of the initial composition of a 50% weathered sample using the ANN and the actual composition. Gasoline sample number: 410.

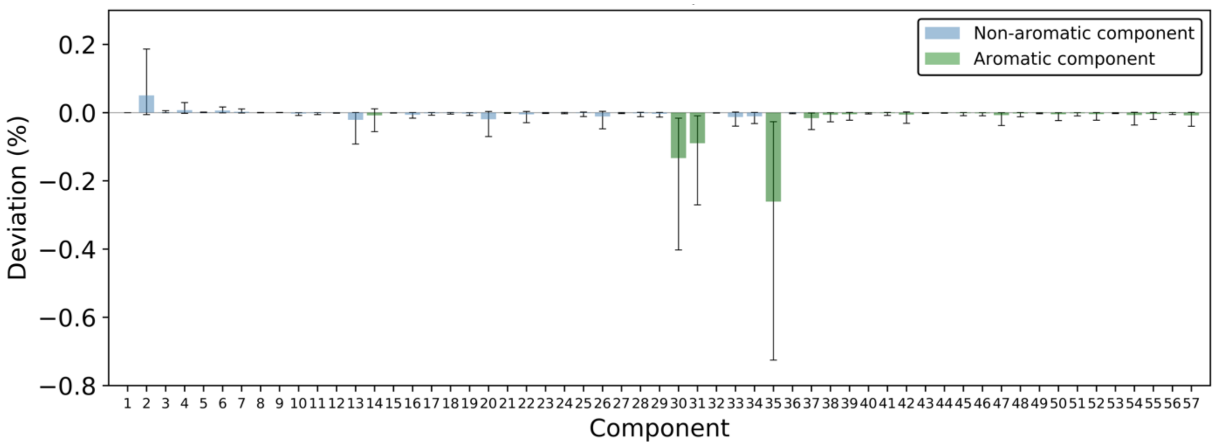

Figure S 17. Prediction of the initial composition of a 50% weathered sample using the ANN and the actual composition. Gasoline sample number: 411.

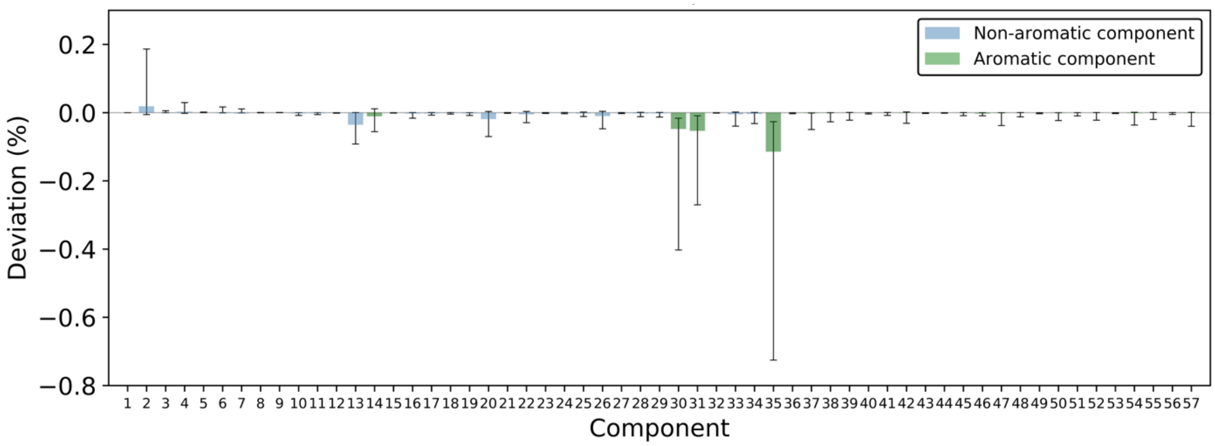

Figure S 18. Prediction of the initial composition of a 50% weathered sample using the ANN and the actual composition. Gasoline sample number: 412.

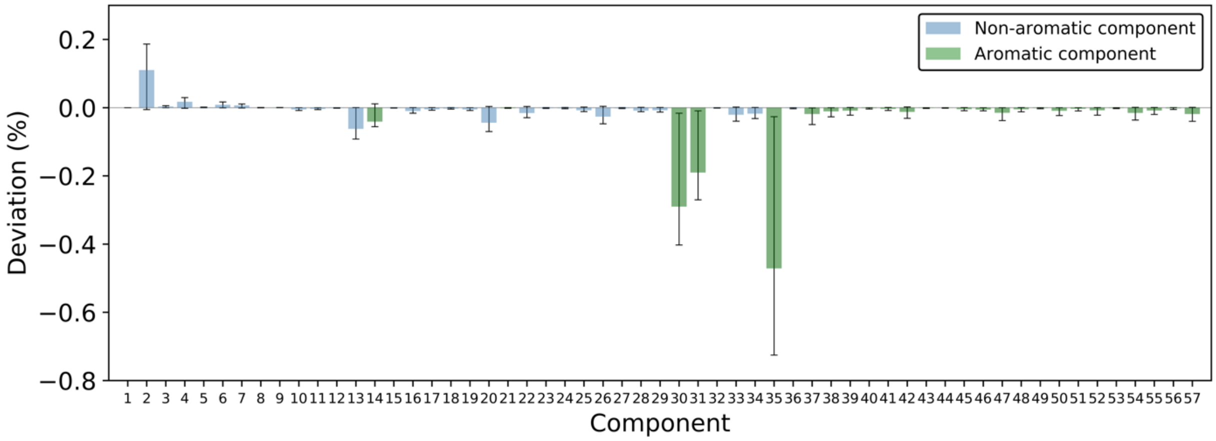

Figure S 19. Prediction of the initial composition of a 50% weathered sample using the ANN and the actual composition. Gasoline sample number: 413.

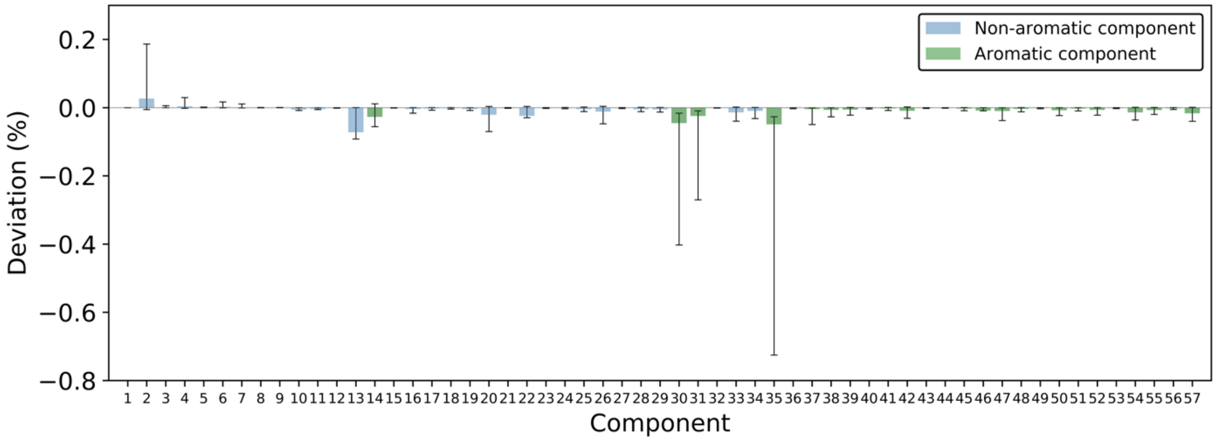

Figure S 20. Prediction of the initial composition of a 50% weathered sample using the ANN and the actual composition. Gasoline sample number: 414.

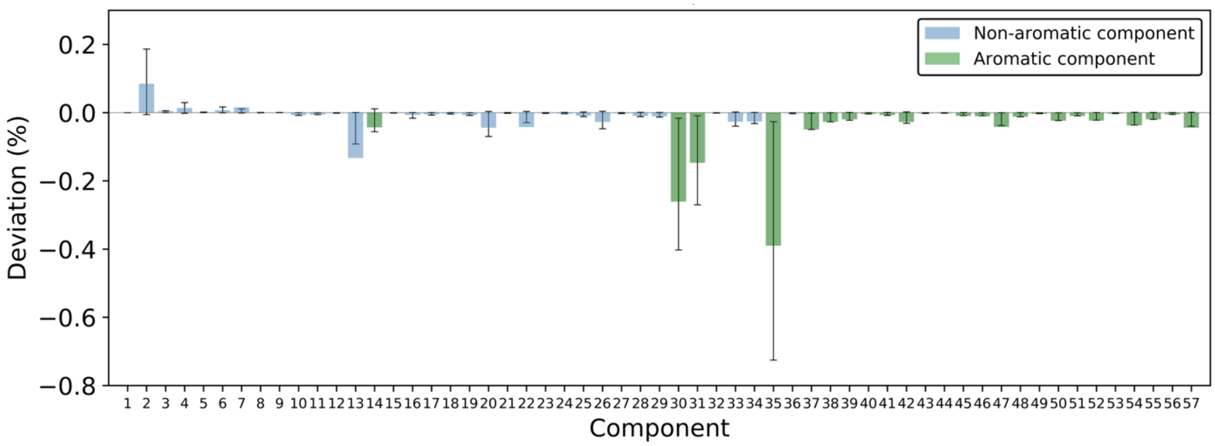

Figure S 21. Prediction of the initial composition of a 50% weathered sample using the ANN and the actual composition. Gasoline sample number: 415.

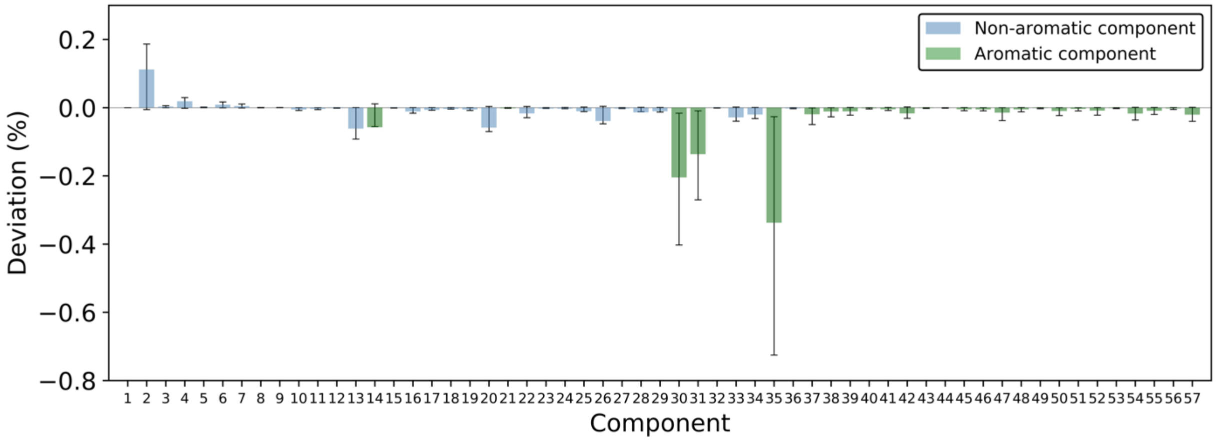

Figure S 22. Prediction of the initial composition of a 50% weathered sample using the ANN and the actual composition. Gasoline sample number: 416.

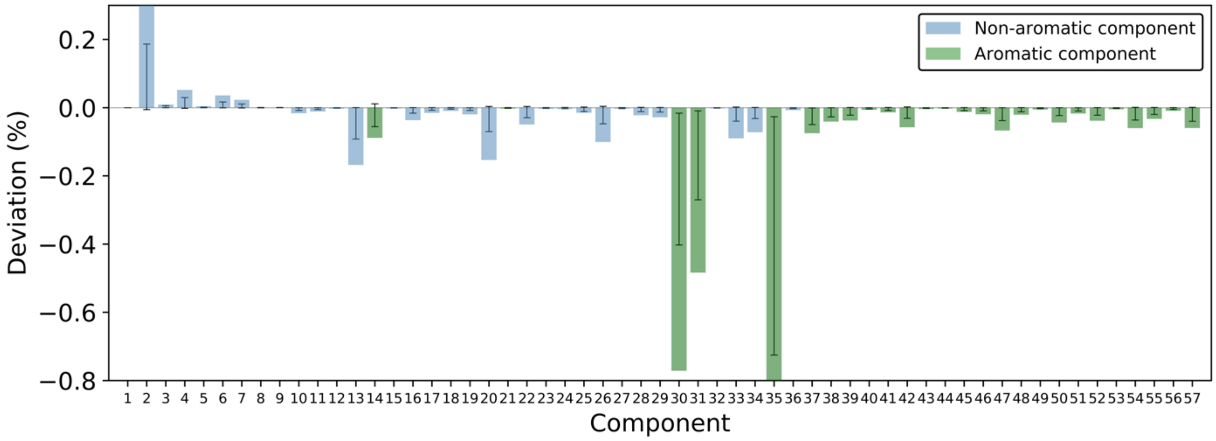

Figure S 23. Prediction of the initial composition of a 50% weathered sample using the ANN and the actual composition. Gasoline sample number: 417.

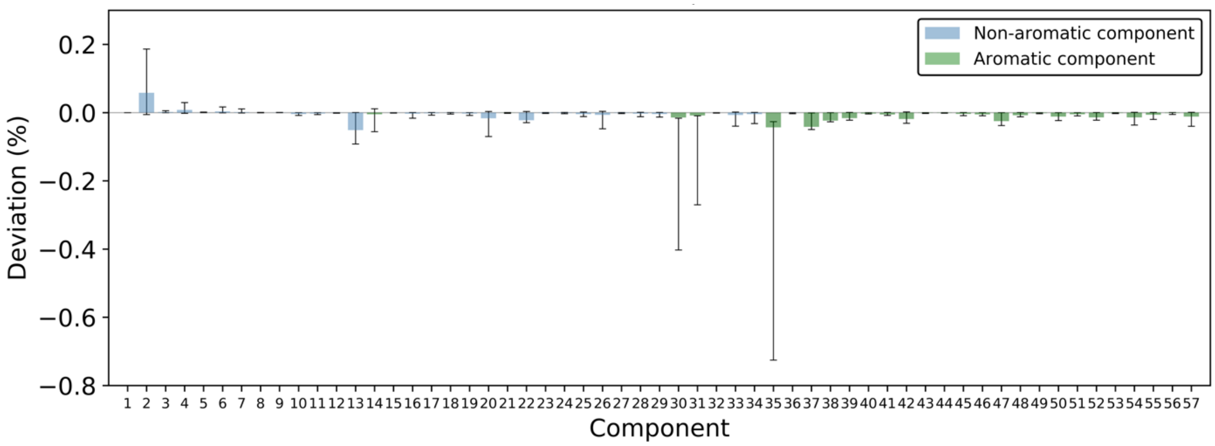

Figure S 24. Prediction of the initial composition of a 50% weathered sample using the ANN and the actual composition. Gasoline sample number: 418.

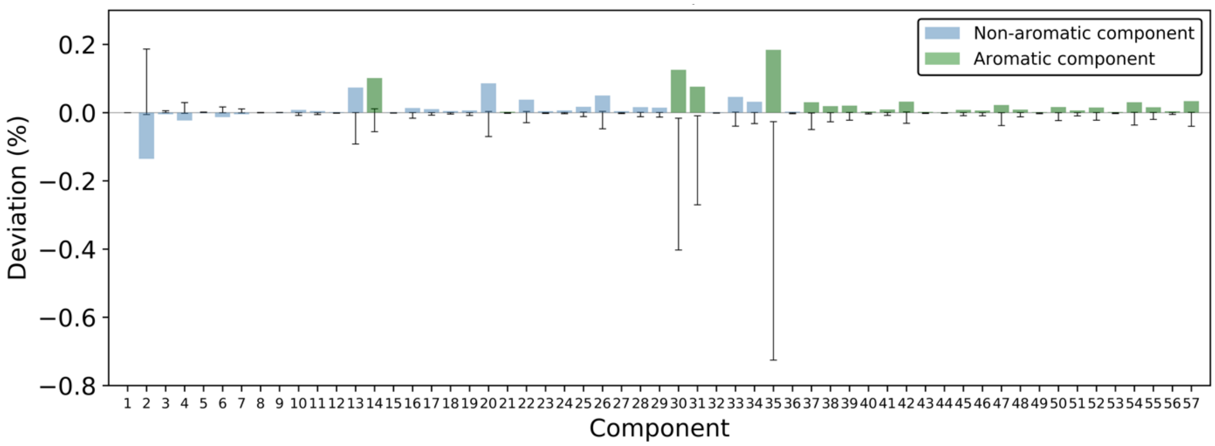

Figure S 25. Prediction of the initial composition of a 50% weathered sample using the ANN and the actual composition. Gasoline sample number: 419.

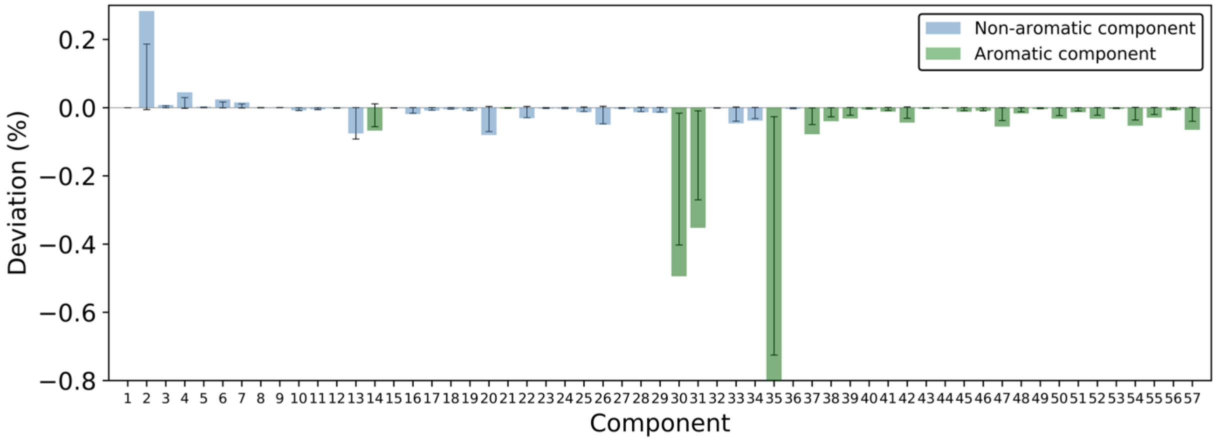

Figure S 26. Prediction of the initial composition of a 50% weathered sample using the ANN and the actual composition. Gasoline sample number: 420.

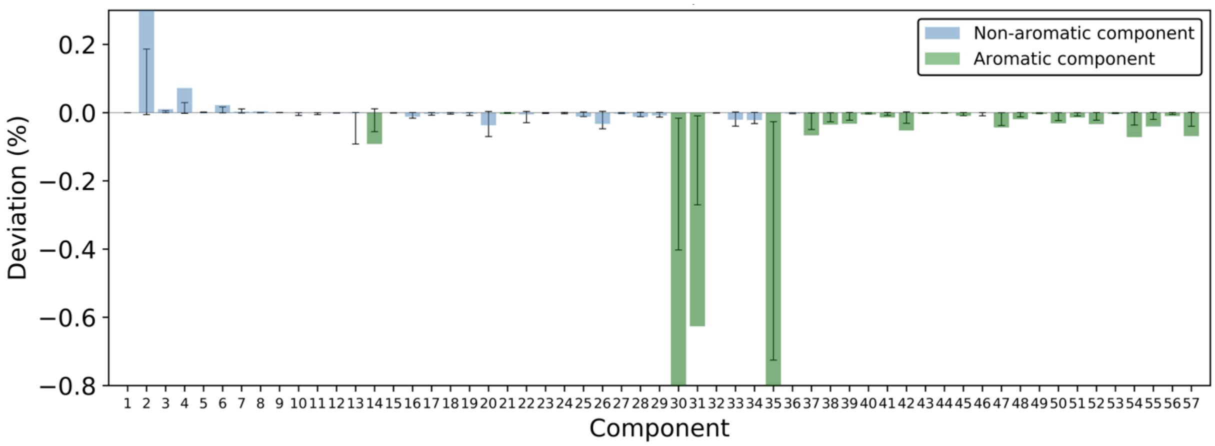

Figure S 27. Prediction of the initial composition of a 50% weathered sample using the ANN and the actual composition. Gasoline sample number: 421.

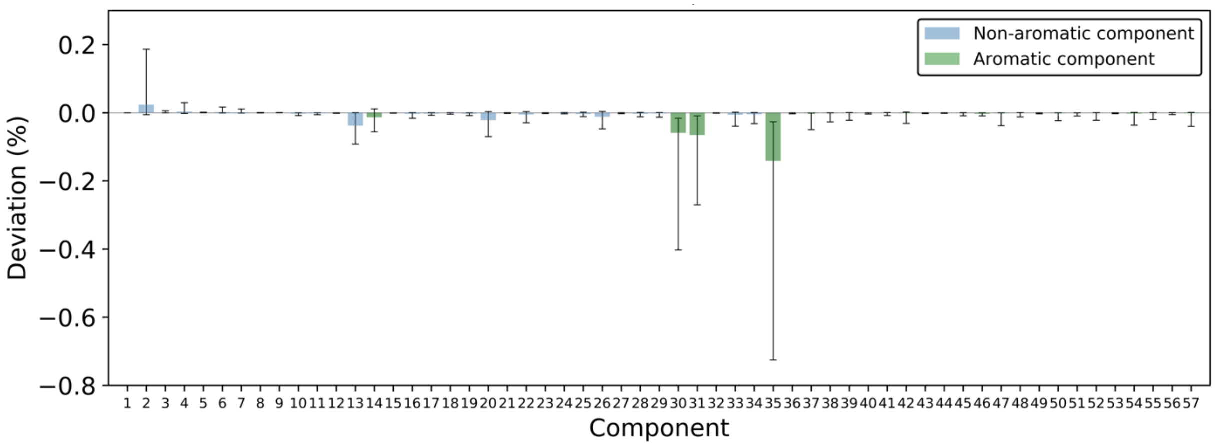

Figure S 28. Prediction of the initial composition of a 50% weathered sample using the ANN and the actual composition. Gasoline sample number: 422.

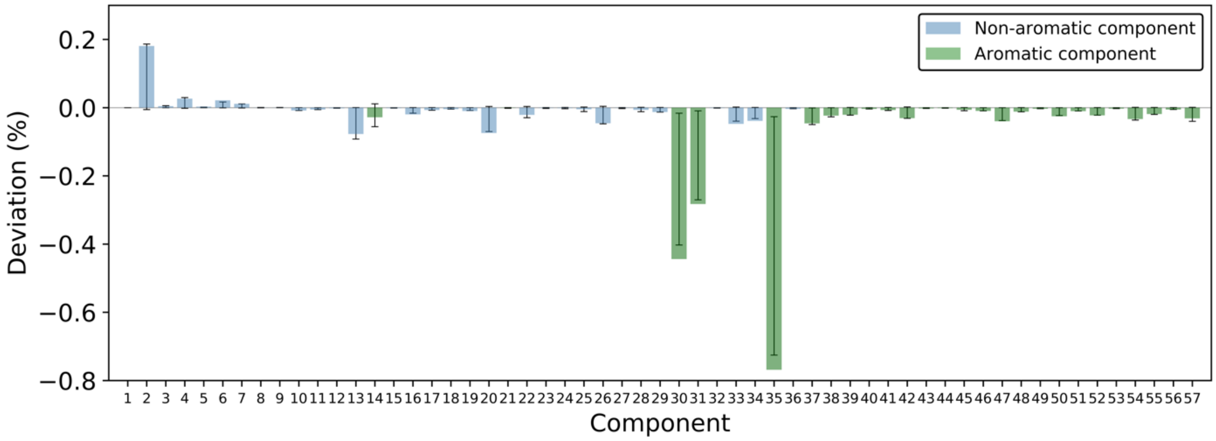

Figure S 29. Prediction of the initial composition of a 50% weathered sample using the ANN and the actual composition. Gasoline sample number: 423.

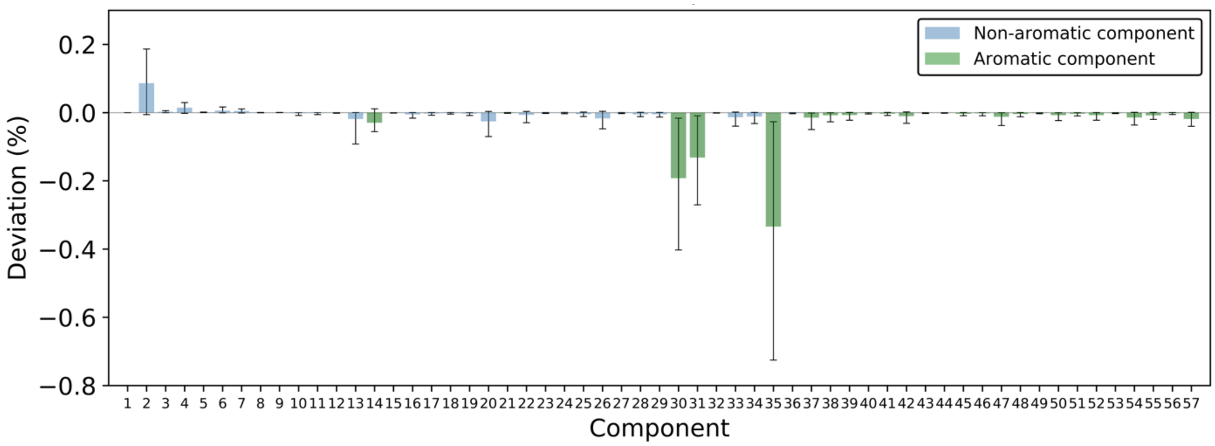

Figure S 30. Prediction of the initial composition of a 50% weathered sample using the ANN and the actual composition. Gasoline sample number: 424.

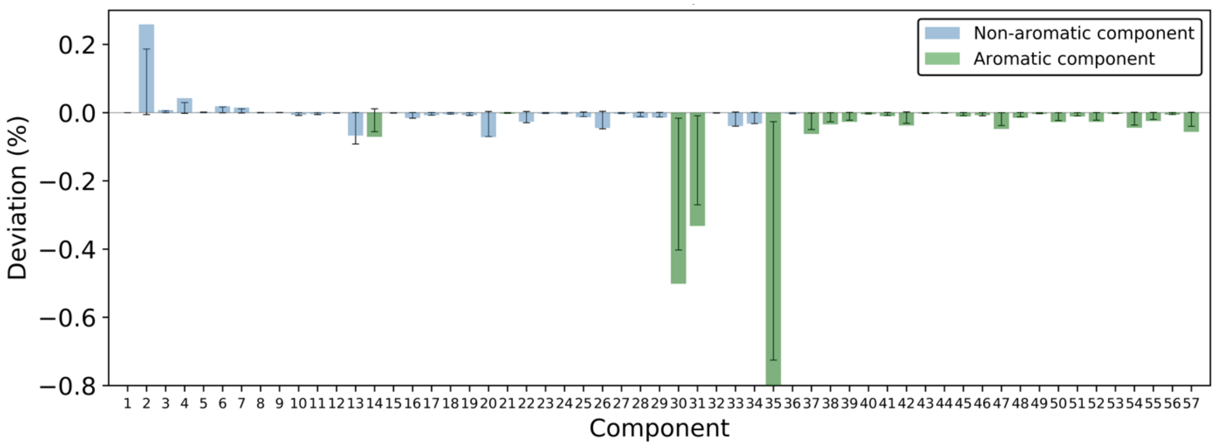

Figure S 31. Prediction of the initial composition of a 50% weathered sample using the ANN and the actual composition. Gasoline sample number: 425.

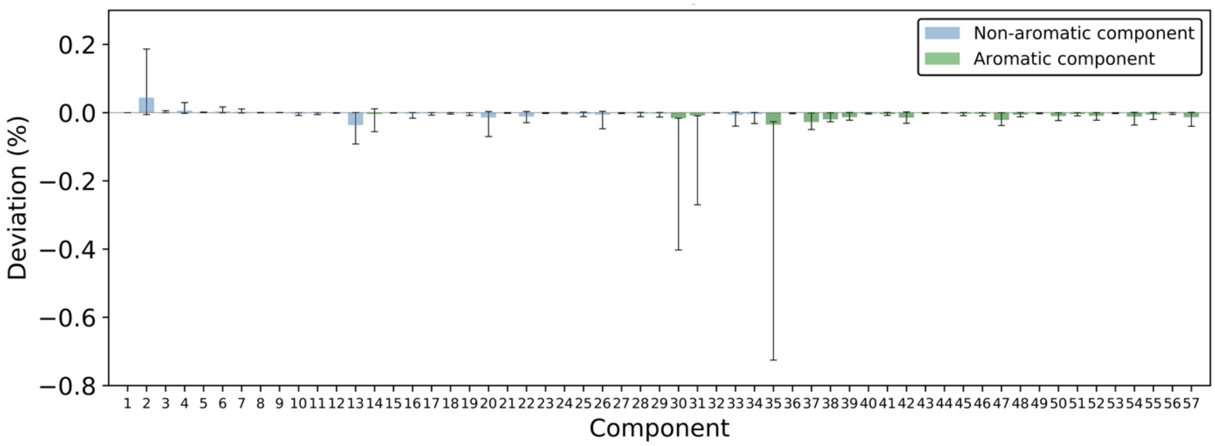

Figure S 32. Prediction of the initial composition of a 50% weathered sample using the ANN and the actual composition. Gasoline sample number: 426.

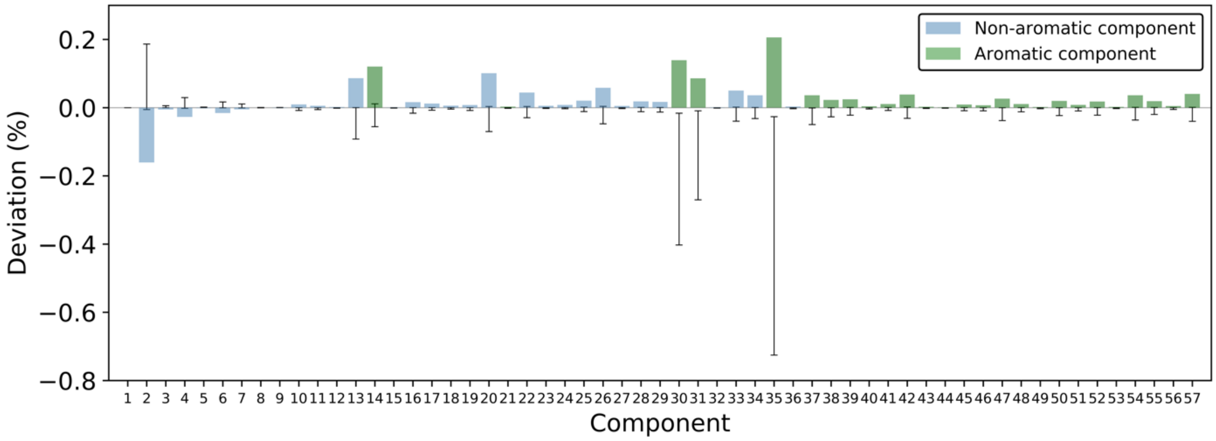

Figure S 33. Prediction of the initial composition of a 50% weathered sample using the ANN and the actual composition. Gasoline sample number: 427.

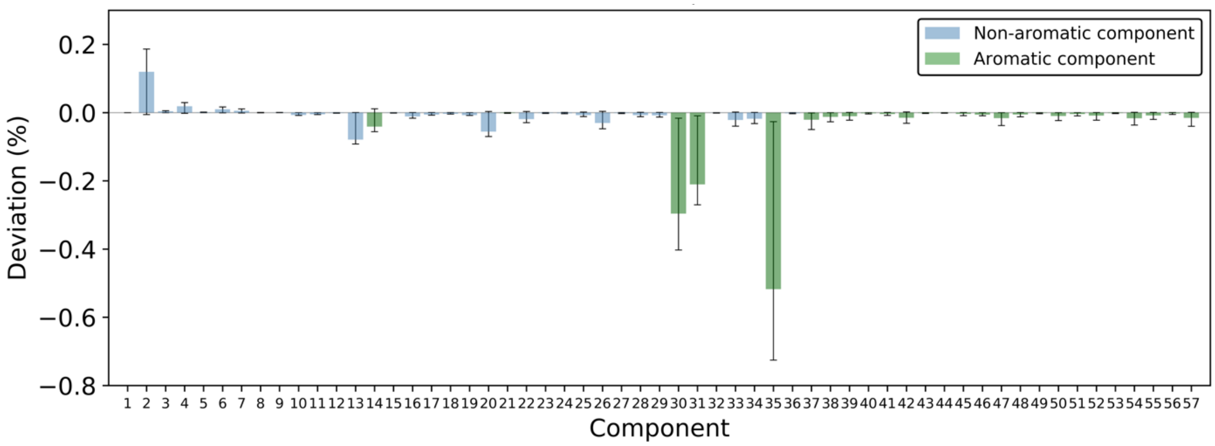

Figure S 34. Prediction of the initial composition of a 50% weathered sample using the ANN and the actual composition. Gasoline sample number: 428.

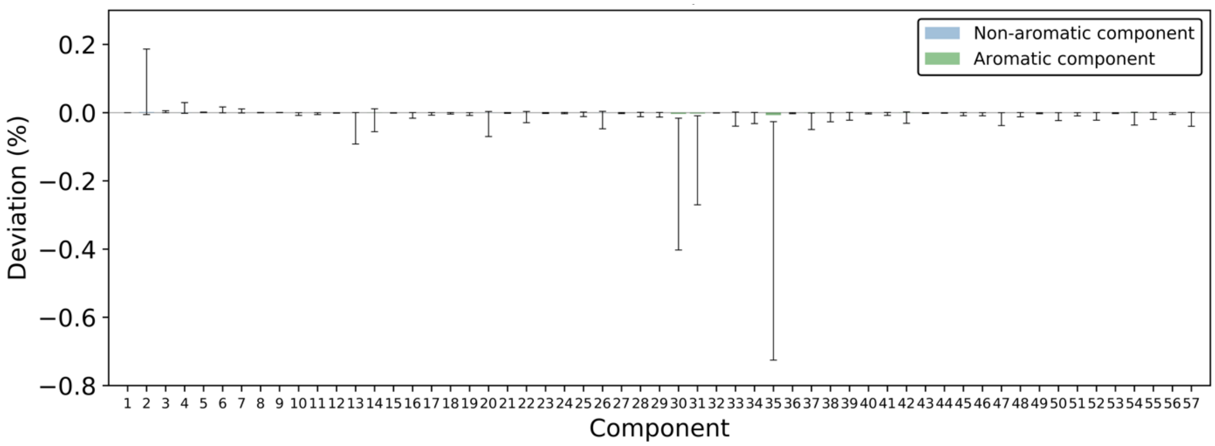

Figure S 35. Prediction of the initial composition of a 50% weathered sample using the ANN and the actual composition. Gasoline sample number: 429.

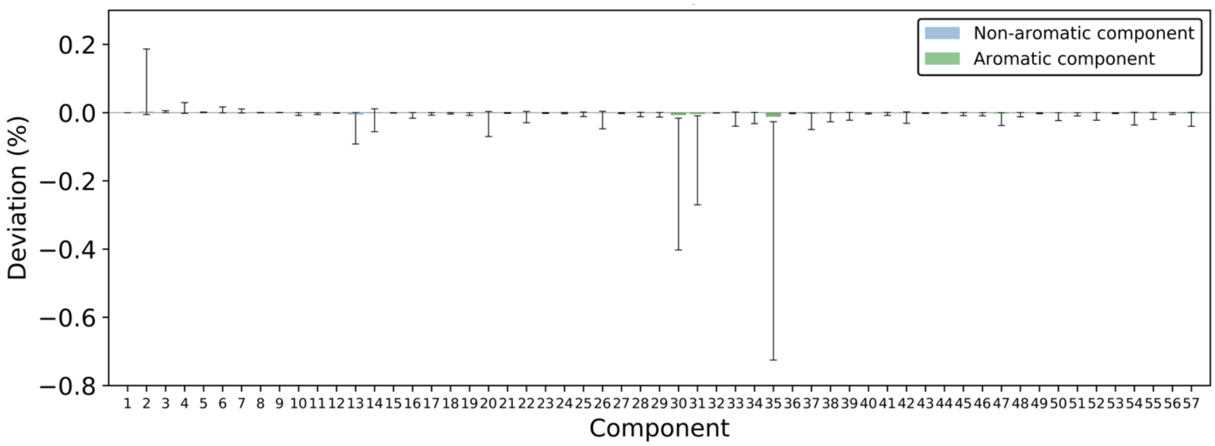

Figure S 36. Prediction of the initial composition of a 50% weathered sample using the ANN and the actual composition. Gasoline sample number: 430.

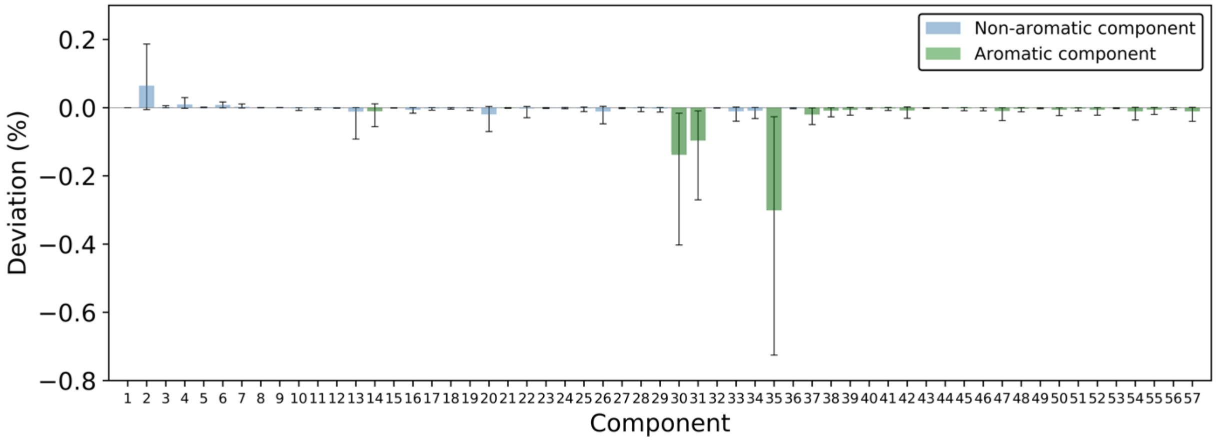

Figure S 37. Prediction of the initial composition of a 50% weathered sample using the ANN and the actual composition. Gasoline sample number: 431.

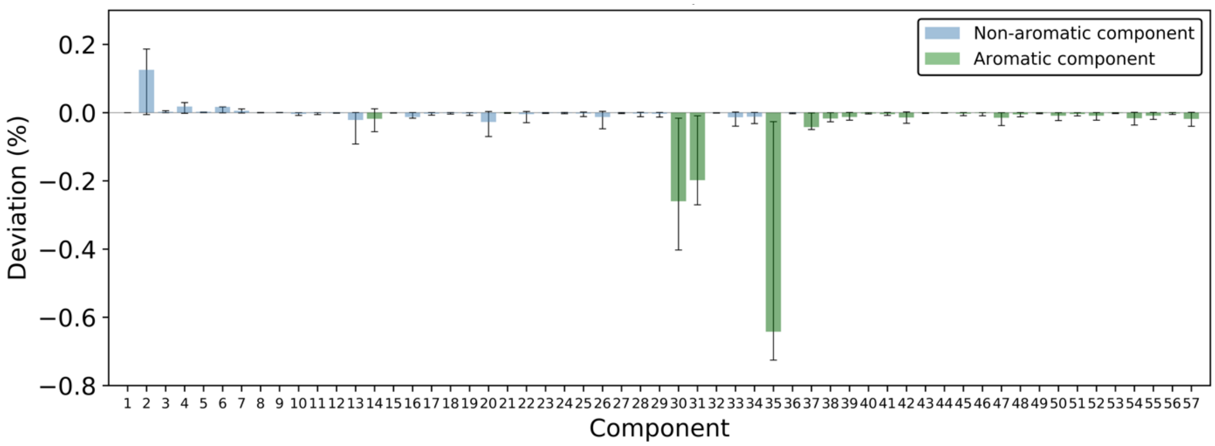

Figure S 38. Prediction of the initial composition of a 50% weathered sample using the ANN and the actual composition. Gasoline sample number: 432.

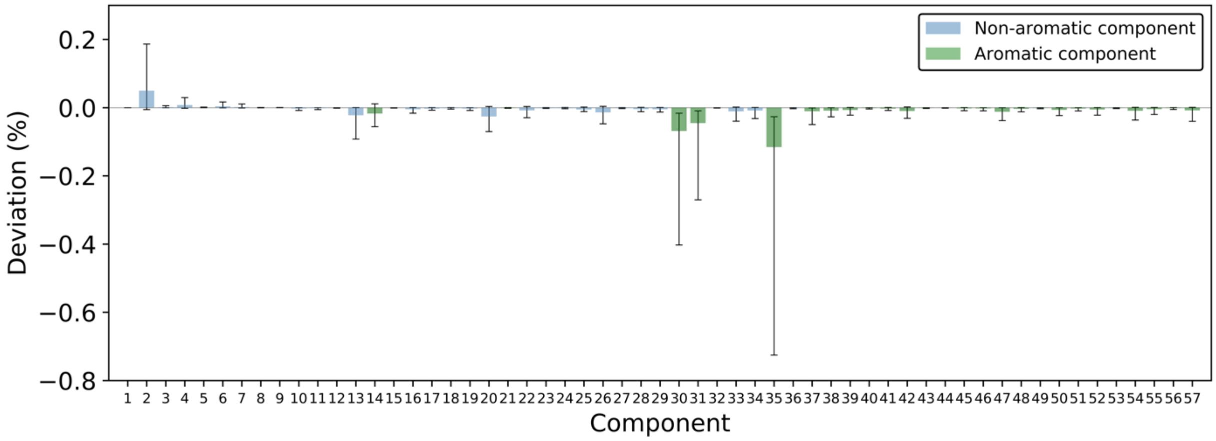

Figure S 39. Prediction of the initial composition of a 50% weathered sample using the ANN and the actual composition. Gasoline sample number: 433.

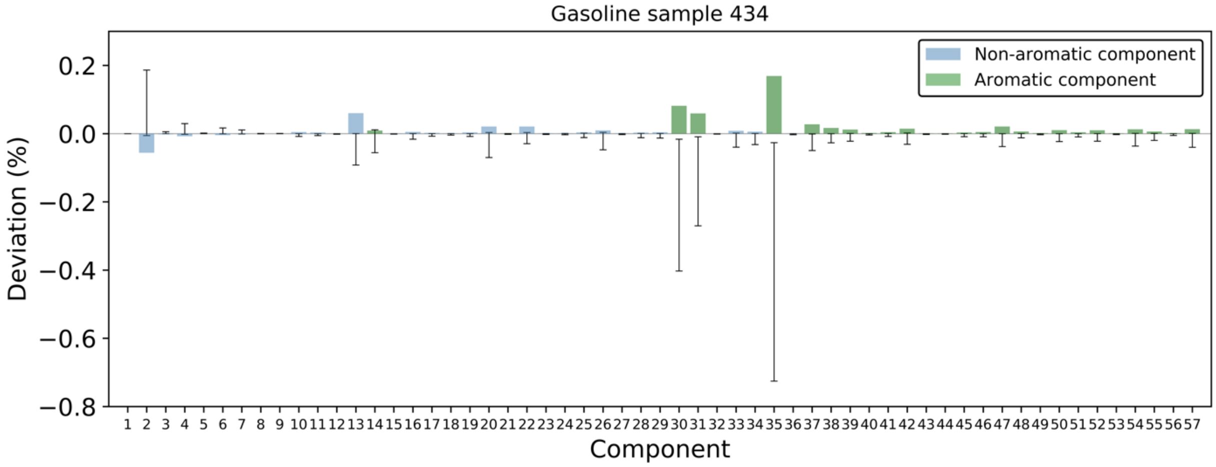

Figure S 40. Prediction of the initial composition of a 50% weathered sample using the ANN and the actual composition. Gasoline sample number: 434.

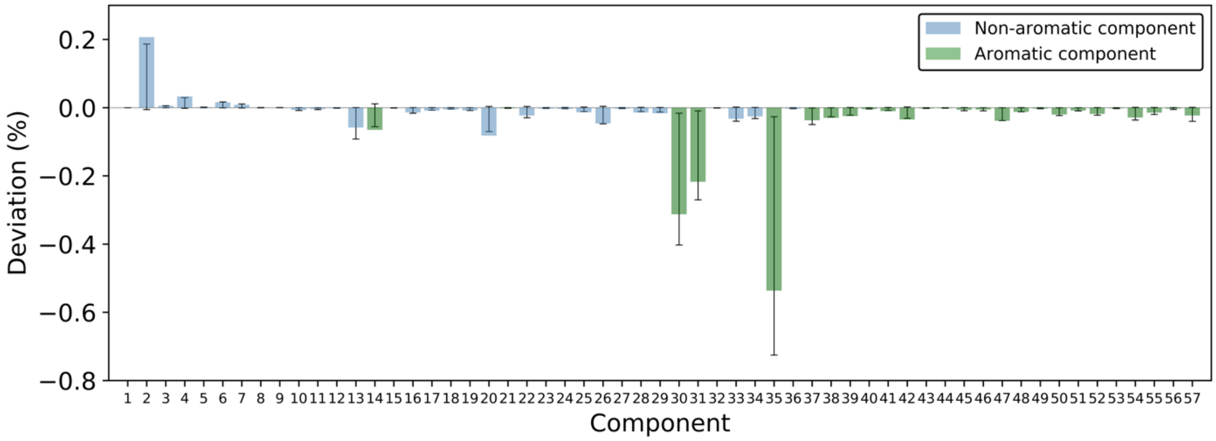

Figure S 41. Prediction of the initial composition of a 50% weathered sample using the ANN and the actual composition. Gasoline sample number: 435.

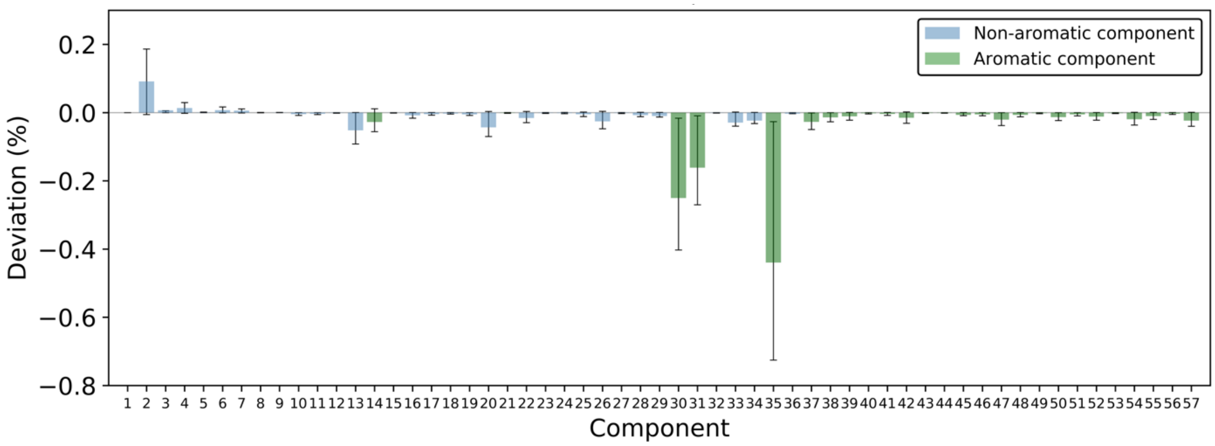

Figure S 42. Prediction of the initial composition of a 50% weathered sample using the ANN and the actual composition. Gasoline sample number: 436.

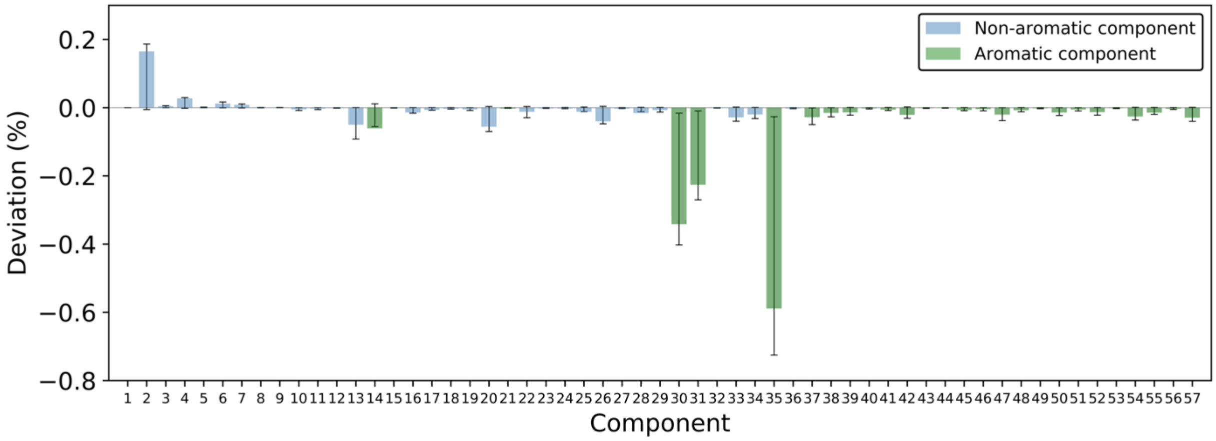

Figure S 43. Prediction of the initial composition of a 50% weathered sample using the ANN and the actual composition. Gasoline sample number: 437.

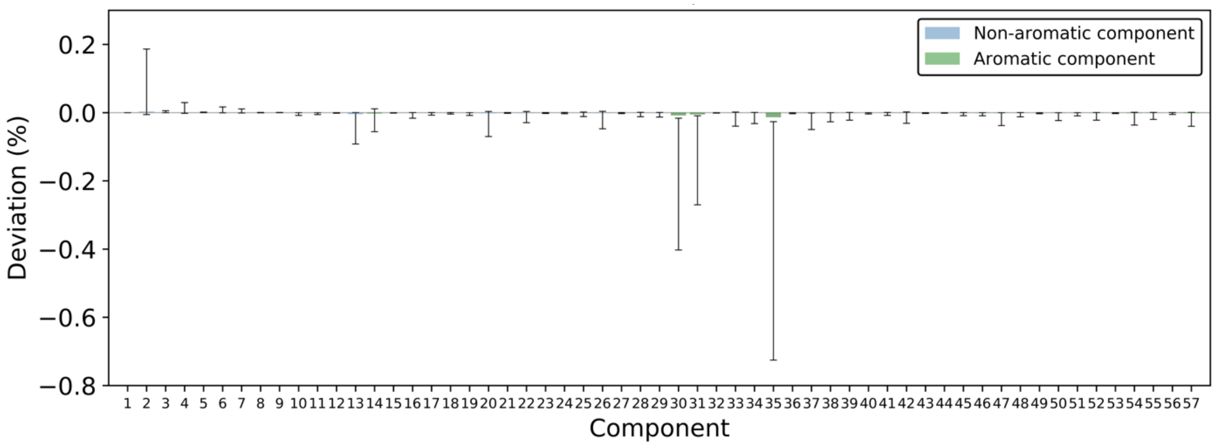

Figure S 44. Prediction of the initial composition of a 50% weathered sample using the ANN and the actual composition. Gasoline sample number: 438.
